# Chronic inhibition of the mitochondrial ATP synthase in skeletal muscle triggers sarcoplasmic reticulum distress and tubular aggregates

**DOI:** 10.1038/s41419-022-05016-z

**Published:** 2022-06-22

**Authors:** Cristina Sánchez-González, Juan Cruz Herrero Martín, Beñat Salegi Ansa, Cristina Núñez de Arenas, Brina Stančič, Marta P. Pereira, Laura Contreras, José M. Cuezva, Laura Formentini

**Affiliations:** 1 Departamento de Biología Molecular, Centro de Biología Molecular ‘“Severo Ochoa’” (CBMSO), c/ Nicolás Cabrera 1, Universidad Autónoma de Madrid, Madrid, Spain; 2 Centro de Investigación Biomédica en red de Enfermedades Raras (CIBERER), ISCIII, Madrid, Spain; 3 Instituto Universitario de Biología Molecular, IUBM, Universidad Autónoma de Madrid, Madrid, Spain; 4 Instituto de Investigaciones Sanitarias Fundación Jiménez Díaz (IIS-FJD), Madrid, Spain; 5 Instituto de Investigación Hospital 12 de Octubre, i+12, Madrid, Spain

**Keywords:** Musculoskeletal abnormalities, Energy metabolism

## Abstract

Tubular aggregates (TA) are honeycomb-like arrays of sarcoplasmic-reticulum (SR) tubules affecting aged glycolytic fibers of male individuals and inducing severe sarcomere disorganization and muscular pain. TA develop in skeletal muscle from Tubular Aggregate Myopathy (TAM) patients as well as in other disorders including endocrine syndromes, diabetes, and ageing, being their primary cause unknown. Nowadays, there is no cure for TA. Intriguingly, both hypoxia and calcium dyshomeostasis prompt TA formation, pointing to a possible role for mitochondria in their setting. However, a functional link between mitochondrial dysfunctions and TA remains unknown. Herein, we investigate the alteration in muscle-proteome of TAM patients, the molecular mechanism of TA onset and a potential therapy in a preclinical mouse model of the disease. We show that in vivo chronic inhibition of the mitochondrial ATP synthase in muscle causes TA. Upon long-term restrained oxidative phosphorylation (OXPHOS), oxidative *soleus* experiments a metabolic and structural switch towards glycolytic fibers, increases mitochondrial fission, and activates mitophagy to recycle damaged mitochondria. TA result from the overresponse of the fission controller DRP1, that upregulates the Store-Operate-Calcium-Entry and increases the mitochondria-SR interaction in a futile attempt to buffer calcium overloads upon prolonged OXPHOS inhibition. Accordingly, hypoxic muscles cultured ex vivo show an increase in mitochondria/SR contact sites and autophagic/mitophagic zones, where TA clusters grow around defective mitochondria. Moreover, hypoxia triggered a stronger TA formation upon ATP synthase inhibition, and this effect was reduced by the DRP1 inhibitor mDIVI. Remarkably, the muscle proteome of TAM patients displays similar alterations in mitochondrial dynamics and in ATP synthase contents. In vivo edaravone treatment in mice with restrained OXPHOS restored a healthy phenotype by prompting mitogenesis and mitochondrial fusion. Altogether, our data provide a functional link between the ATP synthase/DRP1 axis and the setting of TA, and repurpose edaravone as a possible treatment for TA-associated disorders.

## Introduction

First described in 1970 [1], tubular aggregates (TA) are regular arrays of sarcoplasmic reticulum (SR) membranes [2, 3] that form in aged skeletal muscle (Skm) and disrupt sarcomere structure [3]. Interestingly, TA appear almost exclusively in glycolytic fibers [3] and only in males [4, 5]. As a result, Tubular Aggregate Myopathy (TAM; ORPHA:2593; OMIM:160565, 615883) courses with progressive myasthenia, cramps, and muscular pain [1, 5, 6]. Remarkably, TA are not restricted to TAM and appear during aging in myopathies, myasthenic and Stormorken syndromes, and endocrine disorders [3, 5, 6]. Nevertheless, nowadays there is no specific pharmacological treatment for TA.

Although TA origin is unknown, gain-of-function mutations in components of the Store-Operated-Calcium-Entry (SOCE) have been described in TAM subjects [7] and result in calcium overload [8], suggesting a role for an inefficient mitochondrial calcium buffering in this disorder. Indeed, during oxidative phosphorylation (OXPHOS), mitochondria integrate ATP production with metabolism [9], reactive oxygen species (ROS) [10–12], and calcium fluxes [13], maintaining calcium homeostasis. The idea that OXPHOS dysfunctions may participate in TA formation also arise by indirect and observational evidences. Hypoxia or mitochondrial complex IV inhibitor cyanide have been shown to generate TA in rat *EDL* muscles cultured ex vivo [14] and a reduction in respiratory complexes I and IV have been reported in a small cohort of TAM patients [15]. Moreover, TA were observed in comorbidity with multiple mtDNA-mutation myopathies [5, 16] and an overall Skm hyperacidemia, linked to an increase in glycolysis and lactate production, has been described in TAM [17]. Intriguingly, oxidative *soleus* cultured in hypoxia does not develop TA [14], making TA appearance in oxidative muscles an extremely rare event in humans [4], further suggesting a role for mitochondria in preventing TA setting. However, no functional link between OXPHOS dysfunctions and TA has been described so far.

To explore the OXPHOS role in TA formation, we have used a Skm-specific mouse model of long-term ATP synthase inhibition. The chronic overexpression of the ATP synthase inhibitor h_H49_IF1 [18, 19] leads to a metabolic switch towards glycolysis, while inducing mitochondrial fission and mitophagy in aged mice. The resulting h_H49K_IF1-dependent DRP1 over-expression prompts SOCE dysregulation and SR distress, causing TA and myopathy. Consistently, hypoxia induces larger TA upon ATP synthase inhibition in ex vivo muscles, and this effect is reduced by inhibiting DRP1.

Overall, we report that the ATP synthase inhibition is a characteristic feature of TAM, being our model a preclinical tool that mimics the pathology. In this regard, we have used a recently identified mitochondrial enhancer, edaravone [18], to reestablish a healthy phenotype in mice. We show that edaravone mechanism of action is related to a burst in mitochondrial dynamics and biogenesis, thus providing a functional link between OXPHOS and the setting of TA.

## Results

### TAM alters Skm mitochondrial OXPHOS, dynamics, and redox system

**Fig. 1:**
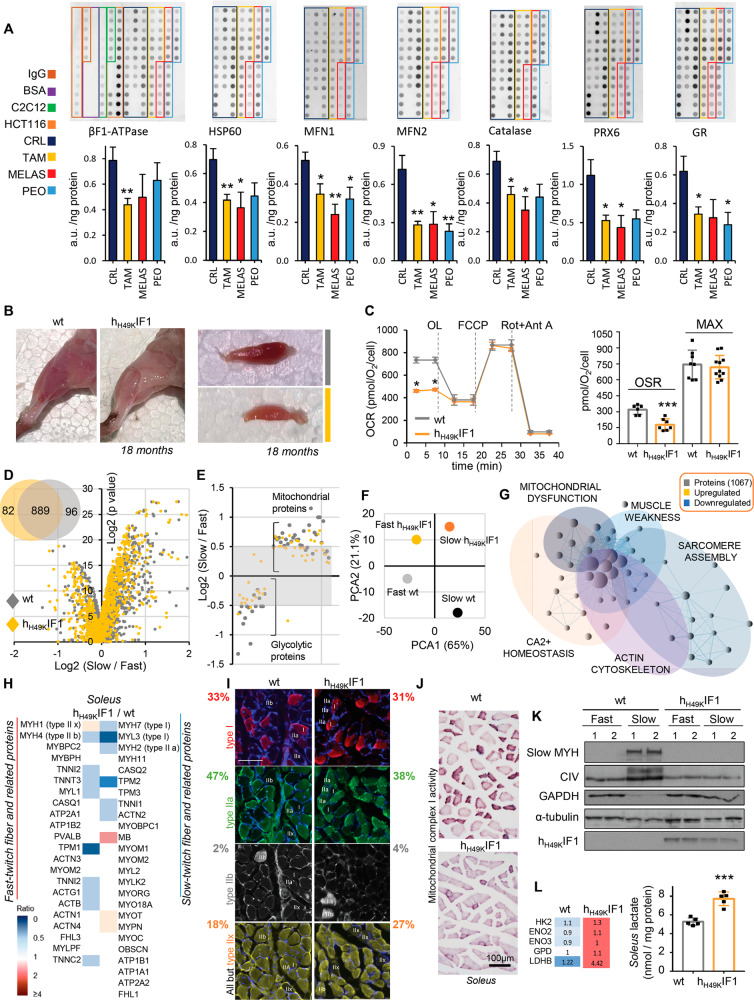
The inhibition of the Skm ATP synthase is a common trait of h_H49K_IF1 mice and TAM patients. **A** Reverse Phase Protein Array (RPPA) of Skm biopsy extracts from healthy (CRL, dark blue; *n* = 11), TAM (yellow; *n* = 6), MELAS (red; *n* = 4) and PEO (light blue, *n* = 4) patients. Linear plot of the mouse muscle C_2_C_12_ cell line was used as loading control. Bars indicate the mean ± SEM of three replicates of the n above indicated. **B** Representative images of hindlimb (left) and *soleus* (right) muscles from 18-months-old wt and h_H49K_IF1 mice. **C** Representative respiratory profile of primary myocytes from wt (gray trace) and h_H49K_IF1 (orange trace) mice. OCR, oxygen consumption rate; OSR: Oligomycin sensitive respiration; MAX: maximal respiration. Orden of injection: 5 μM oligomycin (OL); 5 μM FCCP; 1 μM rotenone (Rot) + 1 μM antimycin A (Ant A). Quantification in right histogram. Bars are the mean ± SEM of three experiments. **D**–**F** Quantitative proteomic analysis (TMT) of *soleus* (slow) and *gastrocnemius* (fast) muscles from wt (*n* = 4) and h_H49K_IF1 (*n* = 4) mice. Volcano plot (**D**), circle chart (**D**) and dot plot (**E**) present the proteins identified in wt (gray) and h_H49K_IF1 (yellow) mice. A (−) log2 *p*-value > 4 was considered statistically significant. **F** Principal component analysis plot (PCA) based on the rank correlation matrix. **G** Cytoscape representation of the GSEA bioinformatic analysis of TMT proteomic data from TMT. Blue-violet or yellow circles represent downregulated or upregulated pathways, respectively, in *soleus* muscle from h_H49K_IF1 mice compared to wt. Exact NES and pathway sizes are reported in Fig. S2B, C. **H** Fast- and slow-twitch markers and related proteins in *soleus*. Higher intensities of red or blue colors represent higher or lower h_H49K_IF1/wt expression ratios, respectively. *n* = 4 animals/genotype. **I** Transversal slices of *soleus* from wt and h_H49K_IF1 18 months-old mice immuno-labeled for antibodies against specific fiber-type MYHs. Red, type I fibers (MYH7, antibody clone: BA-F8); green, type IIa fibers (MYH2, antibody clone: SC-71); gray, type IIb fibers (MYH4, antibody clone: DF-F3); yellow, type IIx fibers (antibody clone: BF-35, all but type IIx fibers). More pictures and details in Fig. S4A, B. wt, *n* = 3; h_H49K_IF1, *n* = 3; 4 images/mouse. **J** Transversal slices of *soleus* from wt and h_H49K_IF1 18 months-old mice stained for mitochondrial complex I activity. wt, *n* = 3; h_H49K_IF1, *n* = 4; 4 images/mouse. **K** Representative WB expression of markers of slow fibers (slow MYH), mitochondria (complex IV), and glycolysis (GAPDH) in *soleus* (slow) and *gastrocnemius* (fast) muscles from wt and h_H49K_IF1. Tubulin is presented as loading control. Two samples per condition, each sample contains protein extracts from 3 mice. Bars are the mean ± SEM of *n* = 6 animals/genotype. **L** Left. Altered proteins from glycolysis in *soleus* (slow) from wt and h_H49K_IF1 animals. Right. *Soleus* lactate levels (wt, *n* = 4; h_H49K_IF1, *n* = 4). *, **, ***p < 0.05; 0.01 and 0.001 when compared to wt by ANOVA and Student’s t-test, respectively. See also Figs. S1–4.

To unveil the connection between Skm OXPHOS and TA, we first searched for Skm mitochondrial dysfunctions in a cohort of TAM patients compared to healthy individuals or subjects that suffer from known mitochondrial myopathies (MELAS, OMIM: 540000; PEO, OMIM: 615084). For this purpose, we used a high-throughput Reverse Phase Protein Array (RPPA) approach [20]. Despite no change in the expression of respiratory complexes II, III, and IV (Fig. S1), TAM Skm biopsies showed a 45% reduction in the ATP synthase catalytic subunit β-F1 ATPase compared to healthy subjects (Fig. 1A). Likewise, the reduced expression of the mitochondrial fusion regulators MFN1 (36%) and MFN2 (61%) (Fig. 1A) suggested alterations in mitochondrial dynamics in TAM. It should be noted that MFNs values were similar to the ones obtained in MELAS and PEO. Mitochondrial dysfunctions alter ROS [10]. Consistently, proteins from the antioxidant response were downregulated in all the myopathies analyzed (Fig. 1A). Other proteins from glucose and FFA metabolism showed no relevant changes (Fig. S1).

Overall, TAM individuals exhibit a compromised mitochondrial bioenergetics, dynamics, and redox homeostasis, being the ATP synthase inhibition a specific trait of the pathology.

### A mouse model recapitulates TAM mitochondrial dysfunctions

We next aimed to investigate whether the observed OXPHOS dysfunction was a cause or consequence of TAM. For this, we relied upon a mouse model of restrained ATP synthase activity, because the genetic manipulation of the β-F1 ATPase content in mammals (as in TAM, Fig. 1A) is incompatible with life [21]. As an alternative, we overexpressed in muscle the ATP synthase’s constitutively active inhibitor, h_H49K_IF1 [18, 19, 22]. Remarkably, 18 months-old mice over-expressing h_H49K_IF1 (Fig. 1B) present a 46% inhibition of the Skm ATP synthase activity (Fig. 1C), reproducing the ATP synthase dysfunction that should occur in TAM as a result of 45% reduction in β-F1 ATPase (Fig. 1A).

Direct observation of hindlimb muscles showed a whitening of fibers upon ATP synthase inhibition (Figs. 1B, S2A), suggesting changes in muscles with chronic mitochondrial dysfunction. We thus sought to unveil the impact of limiting OXPHOS on the Skm proteome by performing TMT-quantitative proteomics on *soleus* and *gastrocnemius* muscles from 18 months-old wt and h_H49K_IF1 mice (Figs. 1D–H, S2B–D). As expected, *soleus* -mainly oxidative- showed significant proteomic differences when compared to the glycolytic *gastrocnemius* in wt animals, characterized by mitochondrial proteins overexpression and glycolytic enzymes downregulation (Figs. 1E, S3). However, a much lower difference was detected between *soleus* and *gastrocnemius* of h_H49K_IF1 mice (Figs. 1E, S1). Accordingly, principal component analysis (PCA) showed no clusters of samples based on their similarity, revealing separations between the 4 muscles analyzed (Fig. 1F).

Gene Set Enrichment (GSEA) bioinformatic analysis of the TMT results revealed *soleus* perturbations in calcium homeostasis, cytoskeleton, and sarcomere assembly pathways upon prolonged OXPHOS inhibition (Figs. 1G, S2B–D). Remarkably, the expression of slow-twitch fiber markers was reduced in *soleus* from h_H49K_IF1 mice (Figs. 1H, S2D), suggesting altered Skm structure.

### The metabolic switch to glycolytic fibers prompts TA setting in *soleus*

Metabolic state influences fiber composition [23, 24]. In order to unveil the fiber-type switch experimented by *soleus* upon chronic ATP synthase inhibition, we next checked on the expression of fiber-type specific Myosin Heavy Chains (MYHs). We observed perturbations in both the RNA (Fig. S2E) and protein expression (Fig. 1H, I) of MYHs and related proteins in 18-months old h_H49K_IF1 compared to wt. In particular, *soleus* cross-sections immuno-labeled with specific MYHs antibodies revealed a decrease in MYH2, a marker of type IIa fibers, and an increase in type IIx and IIb fibers upon ATP synthase inhibition (Fig. 1I and S4A, B). Contrary to what observed in human Skm [25, 26], type IIa fibers present the highest number of mitochondria in mouse, followed by the hierarchy IIx > I > IIb [27–29]. Accordingly, the inhibition of the ATP synthase and a decrease in IIa fibers (Fig. 1I) leads to a reduced mitochondrial complex I (CI) activity in *soleus* sections from h_H49K_IF1 mice (Fig. 1J). To note that in these animals the residual CI activity (darker fibers in Fig. S2F) fully co-localizes with type I and IIx fibers (Fig. S2F), raising the possibility of a metabolic switch in type IIa fibers. These structural changes were also accompanied by the rewiring of the *soleus* glycolytic/oxidative GAPDH/complex IV ratio (Fig. 1K) and resulted in an increased lactate production (Fig. 1L), indicating a switch to a more glycolytic kind of fibers.

**Fig. 2:**
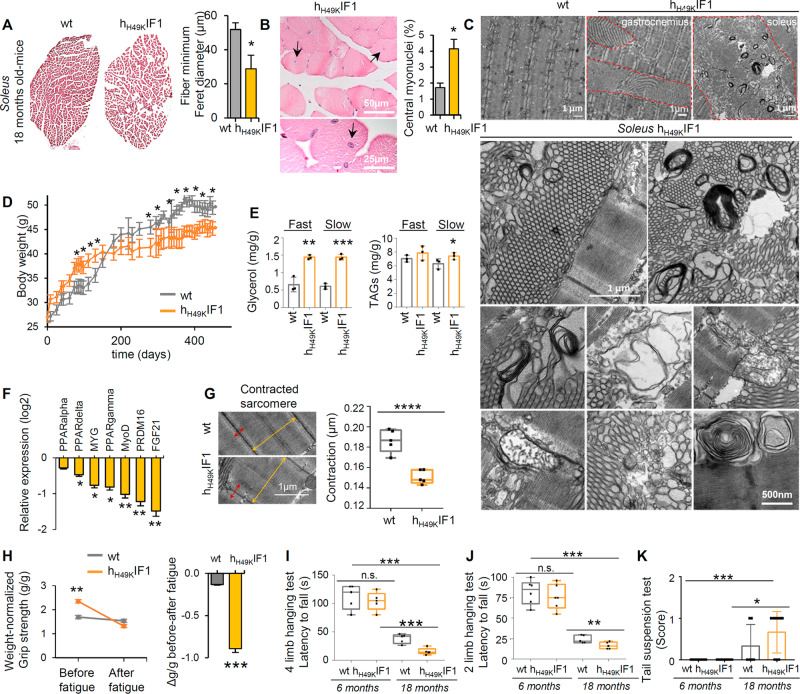
Sarcomere dysregulation and TA in “glycolytic” *soleus* induce myopathy in h_H49K_IF1 mice. **A** Transversal slices of *soleus* from wt and h_H49K_IF1 18 months-old mice stained with haematoxylin/eosin. Images are representative of *n* = 3 mice/genotype. 10 images/mouse. Histograms in the right show fiber minimum Feret diameter. Bars are the mean ± SEM of *n* = 10 measurement/mouse/genotype. **B** Localization of myonuclei in transversal slices of *soleus* from h_H49K_IF1 18 months-old mice stained with haematoxylin/eosin. Histograms show the quantification of central myonuclei per field in 10 images/mouse of both genotypes. See also Fig S4C. **C** TEM images of longitudinal *gastrocnemius* and *soleus* slices from h_H49K_IF1 mice. Extended TA structures, cylindrical spirals, aberrant mitochondria, multilamellar structures, and debris observed upon OXPHOS inhibition. Images are representative of *n* = 4 mice/genotype. 25 images/mouse. **D** Body weight following the Skm expression of h_H49K_IF1. wt, *n* = 12; h_H49K_IF1, *n* = 12. **E** *Soleus* (slow) and *gastrocnemius* (fast) amounts of total glycerol and total triglycerides (TAGs). Bars are the mean ± SEM of *n* = 4 animals/genotype. **F** qPCR relative expression of proteins related to Skm homeostasis. wt, *n* = 4; h_H49K_IF1, *n* = 4. Values are expressed as h_H49K_IF1/wt ratio. Relative expression of Peroxisome Proliferator Activated Receptors (PPARalpha, delta, and gamma); myoglobin, MYG; Myogenic Differentiation 1, MyoD, PRDM16, and the myokine FGF21 is shown. **G** TEM images of contracted sarcomere from wt and h_H49K_IF1 mouse *soleus*. Plots show contraction. Measures are taken in 10 images/mouse. Plots are the mean ± SEM of *n* = 4 animals/genotype. **H**–**K** Motor behavior assays. Grip-force test (**H**) before and after 1 h rotarod fatigue in 18 months old mice (wt, *n* = 7; h_H49K_IF1, *n* = 6). Histograms show the Δ*g*/*g* (force/weight) after-before exercise. Latency to fall in 4 limb (**I**) and 2 limb (**J**) hanging tests in 6 months- and 18 months-old animals. *n* = 5 animals/genotype/age. Tail suspension test (**K**) in 6 months- and 18 months-old. *n* = 3–8 animals/genotype/age. Bars are the mean ± SEM of the reported *n*. In **G**, **I**–**K**, box plots represent 25th to 75th percentiles with the median value in the middle line, and with all data represented from minimal to maximal values. *, **, ***p < 0.05; 0.01 and 0.001 when compared to wt by ANOVA and Student’s t-test, respectively. See also Figs. S2, 4, 5.

In line with this structural and metabolic impairment, whole-*soleus* muscle was compromised when h_H49K_IF1 was expressed (Fig. 2A). Consistent with atrophy and muscle dysfunction, the fiber minimum Feret diameter was smaller (Fig. 2A) and the number of central myonuclei increased (Figs. 2B, S4C) in *soleus* from h_H49K_IF1 mice. Besides, when tissue structure was observed by transmission electron microscopy (TEM), multi-lamellar structures, cylindrical spirals, autophagic vesicles and debris were observed upon ATP synthase inhibition (Fig. 2C), whereas wt animals presented normal aged fiber aspect. It has been reported that TA developed with ageing in almost all inbred mice ^4^ and exclusively in glycolytic fibers [1, 3]. Consistent with this, TA were observed in *gastrocnemius* from both 18 months-old genotypes, although significantly larger when the ATP synthase was inhibited (Figs. 2C, S5A). Remarkably, massive TA structures that were not present in wt *soleus*, appeared in the “glycolytic” *soleus* of 18-months old h_H49K_IF1 mice (Figs. 2C, S5A).

### OXPHOS inhibition results in sarcomere disorganization and myopathy

The h_H49K_IF1-mediated Skm dysfunction determined an overall reduction in mouse weight (Fig. 2D), despite reported rise in lipogenesis in young animals ([18], Fig. S5B) and increased glycerol and TAGs (Fig. 2E). Moreover, gene-expression levels of Skm homeostasis markers (PPAR family, MYG, MyoD, PRDM16, FGF21, Fig. 2F) and proteins from the redox detoxification system (Fig. S5C) were downregulated in *soleus* upon chronic OXPHOS inhibition. Therefore, we next sought to unveil whether sarcomere was affected. We found that chronic ATP synthase inhibition dysregulated the actin-related sarcomere organization (Figs. 2G, S5D–K) by increasing the I band and H zone length compared to wt (Fig. S5J, K). This resulted in a higher sarcomere length in contracted *soleus* (Fig. S5G), indicating an overall lower capacity of fiber contraction upon OXPHOS inhibition (Fig. 2G). Moreover, extended cell death debris zones due to aberrant SR formations and TA were observed in *soleus* from h_H49K_IF1 mice (Figs. 2C, S6A), what ultimately resulted in sarcomere disorganization.

In order to investigate if the sarcomere disruption induces animal motor dysfunction similar to TAM patients, 6- or 18-months old wt and h_H49K_IF1mice were tested for motor behavior (Fig. 2H–K). In agreement with previous findings [30], aged mice of both genotypes had significantly reduced latency to fall and lower muscular force when compared to young mice (Fig. 2H–K). Our first approach was to perform a grip force test before and after rotarod induced fatigue (Fig. 2H). Surprisingly, 18-months old h_H49K_IF1 mice performed better than wt on this test before fatigue (Figs. 2H, S5L). However, while wt maintained almost the same strength capacity after 1 h rotarod workout, the inhibition of the ATP synthase induced a drop in mouse performances after exercise (Figs. 2H, S5L). Similarly, mice with long-term restrained OXPHOS had significantly reduced latency to fall in four (Fig. 2I) and two (Fig. 2J) limb hanging tests and obtained worst performances in tail suspension test (Fig. 2K), suggesting dyskinesia and muscle weakness.

### “Glycolytic” *soleus* shows increased mitochondrial fission and mitophagy

**Fig. 3:**
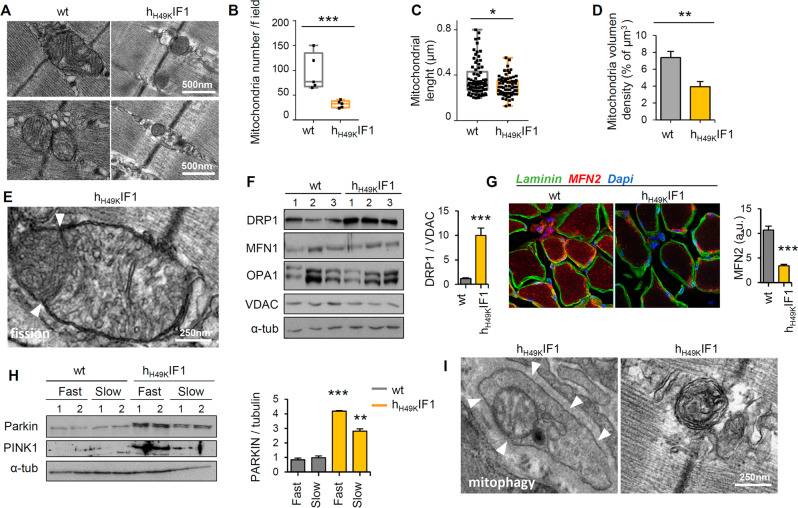
Perturbations in mitochondrial dynamics and mitophagy upon chronic ATP synthase inhibition. **A**, **E**, **I** TEM images of Skm mitochondria in longitudinal *soleus* slices from wt and h_H49K_IF1 mice. Mitochondrial fission and mitophagy were observed in h_H49K_IF1 Skm. Arrowheads point to undergoing fission (**E**) or mitophagy (**I**). *n* = 4 mice/genotype. 25 images/mouse. Quantification of mitochondrial number per field (**B**), diameter (**C**), and volume density (**D**) in longitudinal *soleus* slices from wt and h_H49K_IF1 mice. Measures are taken in *n* = 4 animals/genotype, 10 images/mouse. **F** Representative WB expression of proteins related with mitochondrial dynamics in *soleus* from wt h_H49K_IF1 mice. DRP1, dynamin-1-like; MFN1, mitofusin 1; OPA1, Optic Atrophy 1, and VDAC proteins are shown. Tubulin as loading control. Three samples per condition, each sample contains protein extracts from 3 mice. DRP1 quantification related to VDAC is shown in the below histogram. Bars are the mean ± SEM of *n* = 6 animals/genotype. **G** Immunofluorescence from transversal slices of *soleus* from wt and h_H49K_IF1 18 months-old mice. Green, laminin; red, MFN2; blue, Dapi. MNF2 quantification in the histograms on the right. Bars are the mean ± SEM of *n* = 3 mice/genotype; 10 images/mouse. **H** Representative WB expression of proteins related with mitophagy in *soleus* (slow) and *gastrocnemius* (fast) muscles from wt and h_H49K_IF1 mice. Parkin, E3 Ubiquitin-Protein Ligase and PINK1, PTEN Induced Kinase 1 proteins are shown. Tubulin as loading control. 2 samples per condition, each sample contains protein extracts from 3 mice. Parkin quantification related to tubulin is shown in the right histogram. Bars are the mean ± SEM of *n* = 6 animals/genotype. In **B**–**D**, box plots represent 25th to 75th percentiles with the median value in the middle line, and with all data represented from minimal to maximal values. *, **, ***p < 0.05; 0.01 and 0.001 when compared to wt by ANOVA and Student’s t-test, respectively.

We next tested possible modifications in mitochondrial structure related to the observed phenotype. TEM imaging of *soleus* revealed changes in the shape (Fig. 3A), number (Fig. 3B), length (Fig. 3C) and volume density (Fig. 3D) of mitochondria in h_H49K_IF1 compared to wt littermates. Consistent with a tight cross-talk among OXPHOS, ROS, and mitochondrial dynamics [31, 32], alterations in the redox system were observed upon ATP synthase inhibition (Fig. S5C), and mitochondria appeared smaller, circular, and fissioned (Fig. 3A, E). In line with this and what observed in TAM (Fig. 1A), the mitochondrial fission regulator Dynamin-Related Protein 1 (DRP1) was over-expressed (Fig. 3F) and MFN2 significantly downregulated (Fig. 3G) in *soleus* from h_H49K_IF1 mice compared to wt. It is known that mitochondrial fission facilitates mitophagy [33]. Notably, an increase in the mitophagic regulators PINK1 and parkin was observed in h_H49K_IF1 (Fig. 3H), suggesting that long-term OXPHOS dysfunction may stimulate fission (Fig. 3E) and mitophagy (Fig. 3I) as an attempt of recycling damaged mitochondria.

### ATP synthase/DRP1 axis drives SOCE hyper-activation, SR distress, and TA

**Fig. 4:**
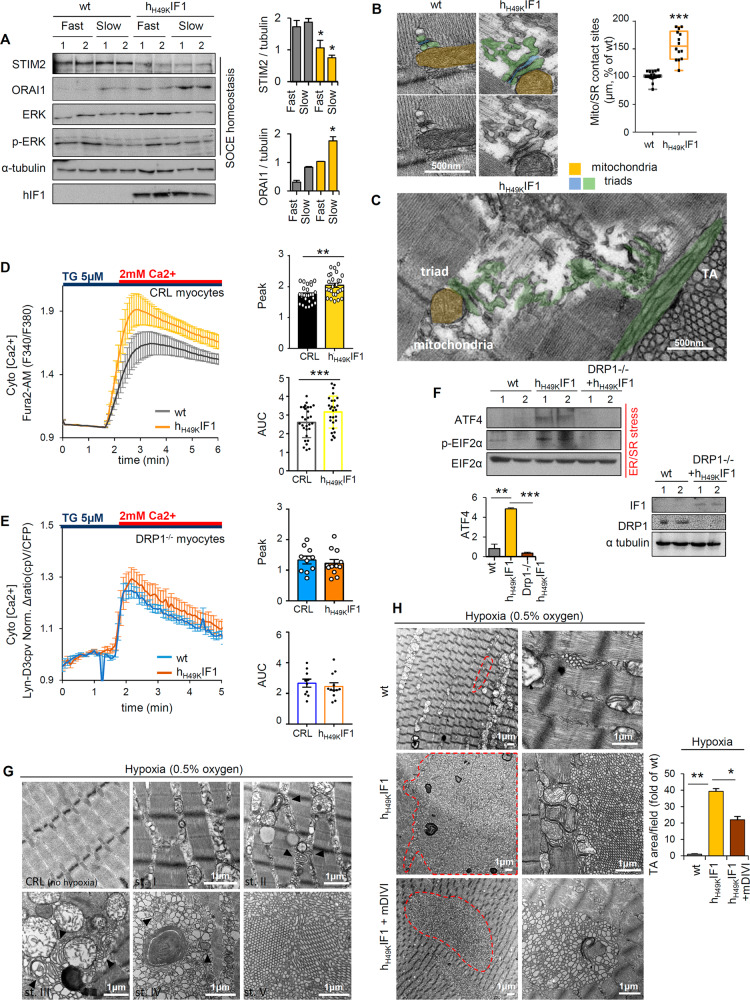
The ATP synthase/DRP1 axis dysregulates calcium fluxes and induces TA. **A** Representative WB expression of proteins related with SOCE in *soleus* (slow) and *gastrocnemius* (fast) muscles from wt and h_H49K_IF1 mice. STIM2, ORAI1, ERK, and its phosphorylated state p-ERK proteins are shown. Tubulin is presented as loading control. Two samples per condition, each sample contains protein extracts from 3 mice. Quantification in the right histograms. Bars are the mean ± SEM of *n* = 6 animals/genotype. **B**, **C** TEM images of Skm SR-mitochondria interactions in longitudinal *soleus* slices from wt and h_H49K_IF1 mice. Triads (terminal cisternae, green; T-tubule, blue) and mitochondria (yellow) are shown. Images are representative of *n* = 4 mice/genotype; 10 images/mouse. Cytosolic calcium uptake measured in control (**D**) or DRP1 −/− (**E**) C_2_C_12_ myocytes expressing or not h_H49K_IF1. Curves are the mean ± SEM of three experiments, *n* = 10 cells/genotype/experiment. Histograms below represent Peak and Under Curve Area (AUC) in control (CRL) and h_H49K_IF1 expressing myocytes. **F** Representative WB expression of proteins related with ER/SR in control (CRL), h_H49K_IF1, and h_H49K_IF1- DRP1 −/− myocytes. ATF4, EIF2α, and the phosphorylated state of EIF2α (p-EIF2α) are shown. Two samples per condition; quantification in the right histograms. Bars are the mean ± SEM of 3 experiments. **G**, **H** Representative TEM images of ex vivo muscles after hypoxia. **G** Five TA developing stages (st I–V) were identified. Black arrows in st II, III, and IV point mitochondria adjacent to SR tubules. TA appeared to generate in close contact with dysfunctional mitochondria until organelles are surrounded and degraded (st IV) and only SR tubules remain (st V). **H** Red lines delimitate the TA area. Quantification of TA area per field in right histograms. Bars are the mean ± SEM of: *n* = 4 muscle/genotype; 10 images/mouse. *, **, ***p < 0.05; 0.01; 0.001 when compared to wt by ANOVA and Student’s t-test, respectively. See also Figs. S5, 6.

Dysfunctional mitochondria perturb calcium fluxes [34–36]. Nevertheless, the molecular mechanism by which mitochondria modulates SOCE is still obscure. Of note, the chronic inhibition of the ATP synthase alters the expression of SOCE components (Figs. 4A, S6B). Thus, we next asked whether h_H49K_IF1 might be directly involved in two necessary events for TA onset: the calcium release units (CRUs or triads) disorganization and the SOCE upregulation [7, 37].

In healthy adult striated muscle, triads, located in proximity of the sarcomere A-I band junction, and mitochondria, located adjacent to calcium stores at the Z line, are tethered ([38], Fig. 4B). An increase in the mitochondria/SR contact sites (Fig. 4B) and aberrations in the triad structure have been detected in *soleus* from h_H49K_IF1 mice (Figs. 4B, S6A). Intriguingly, the aberrant SR directly connected mitochondria with TA (Figs. 4C, S6A). To determine if these morphological changes altered the SOCE activity, calcium levels were followed in living myocytes (Fig. 4D). Similarly to what observed in TAM patients-derived myotubes [39], the ATP synthase inhibition increased the SOCE-mediated calcium uptake (Fig. 4D), suggesting a link between mitochondrial dysfunction and calcium overload. This was also accompanied by a slight decrease in mitochondrial calcium uptake (Fig. S6C) with no changes in mitochondrial calcium retention capacity (CRC) (Fig. S6D).

Although, in agreement with literature, the SOCE-dependent spike in calcium was lower in the absence of DRP1 [40] (Fig. 4E), no differences in SOCE activity were observed in wt and h_H49K_IF1 DRP1-null myocytes (Fig. 4E). These data suggest that the ATP synthase-mediated DRP1 overexpression could mediate the calcium dysregulation, what might drive SR distress and TA. Accordingly, knocking out DRP1 rewires the ATF4-mediated ER/SR stress [41] observed in myocytes when the ATP synthase is inhibited (Fig. 4F).

Hypoxia has been described to induce TA in *EDL* and *gastrocnemius* cultured ex vivo [14]. We identified 5 stages in TA setting under hypoxia (Figs. 4G, S6F). Remarkably, although stage V was characterized by severe tissue disorganization and no other structures than SR tubules were detected, early stages involved increased mitochondria/SR contact sites and autophagic/mitophagic zones (Figs. 4G, S6F). In particular, TA clusters appeared to grow around defective mitochondria (Figs. 4G, S6F, states II and III) until they are surrounded and degraded (Figs. 4G, S6F, state IV). Interestingly, under hypoxia the inhibition of the ATP synthase leaded to larger TA than in wt *gastrocnemius* (Fig. 4H). The treatment with the DRP1-inhibitor mDIVI (Fig. S6E) significantly reduces the TA cluster in h_H49K_IF1 muscles (Fig. 4H), thus suggesting a functional link between the ATP synthase/DRP1 axis and the onset of TA.

### In vivo edaravone treatment restores Skm homeostasis

**Fig. 5:**
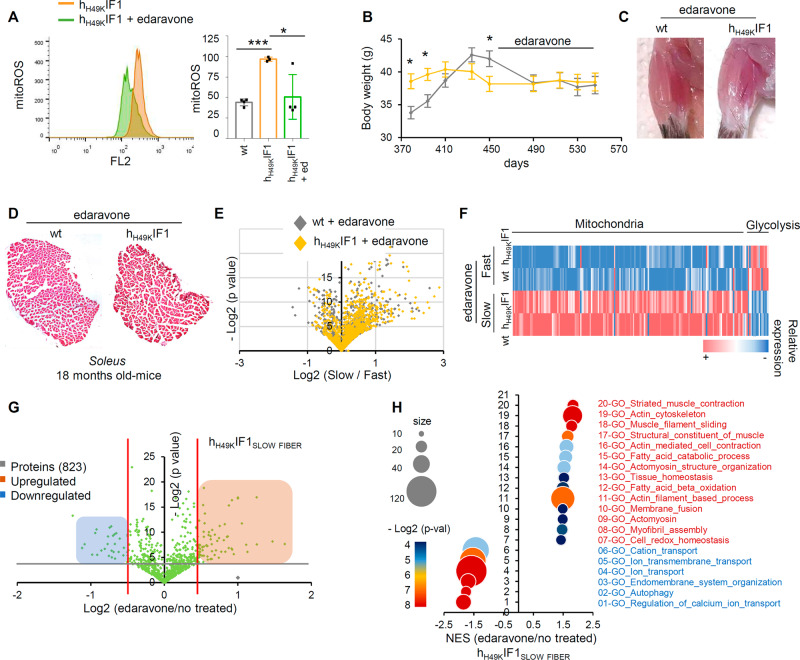
Edaravone rewires Skm and mitochondrial perturbations. **A** MitoSox staining in myocytes expressing or not h_H49K_IF1 and treated with 2 µM edaravone for 24 h. The right histogram shows the quantification of mitochondrial ROS. Bars are the mean ± SEM of *n* = 3 experiments. **B** Body weight graph following edaravone treatment (3 mg/kg) in wt (gray trace) and h_H49K_IF1 (yellow trace) mice (*n* = 5; h_H49K_IF1, *n* = 5). **C** Representative images of *soleus* muscle from wt and h_H49K_IF1 mice. **D** Haematoxylin/eosin stain in transversal slices of *soleus* from wt and h_H49K_IF1 18 months-old mice after 2-months edaravone administration. Images are representative of *n* = 5 mice/genotype. 10 images/mouse. **E**–**G** Quantitative proteomic analysis (TMT) of *soleus* (slow) and *gastronecmius* (fast) muscles from wt (*n* = 4) and h_H49K_IF1 (*n* = 4) mice after 2-month edaravone treatment. Volcano plot (**E**) and heat-map (**F**) of overexpressed (pink) and downregulated (blue) proteins. A (–) log2 *p*-value > 4 was considered statistically significant. In **G**, volcano plot of *soleus* proteins from h_H49K_IF1 mice treated (*n* = 4) or not (*n* = 4) with edaravone. A (–)log2 *p*-value > 4 was considered statistically significant. **H** Bioinformatic analysis of TMT proteomics. Normalized enrichment score (NES) dot chart of 20 perturbed pathways from GSEA bioinformatics analysis. Dot color and size represent *p*-values and the number of altered proteins in the pathway, respectively. *, **, ***p < 0.05, 0.01 and 0.001 when compared to wt by ANOVA and Student’s t-test, respectively. See also Figs. S7, 8.

Finally, we aimed to test whether boosting Skm mitochondria is sufficient to restore Skm homeostasis. Edaravone [42] has been recently identified as a mitochondrial antioxidant and enhancer [18]. Accordingly, edaravone-treated myocytes displayed a higher mitochondrial respiration and lower ROS ([18], Fig. 5A). Two months of in vivo edaravone treatment reduced the animals body weight (Fig. 5B), consistent with its effect on limiting lipid synthesis [18]. Notably, no differences in weight (Fig. 5B) and *soleus* aspect (Fig. 5C, D) were observed between wt and h_H49K_IF1mice after edaravone administration, suggesting a beneficial effect of increasing mitochondrial activity on Skm physiology.

To understand the impact of edaravone on Skm, we performed TMT on *soleus* and *gastrocnemius* from edaravone-treated mice (Figs. 5E–H, S7, S8A). After 2 months of edaravone, the proteome of wt and h_H49K_IF1 animals was similar (Fig. 5E) and characterized by the physiological overexpression of mitochondrial proteins in *soleus* and glycolytic enzymes in *gastrocnemius* (Figs. 5F, S7). The GSEA bioinformatic analysis of treated vs no treated h_H49K_IF1 *soleus* (Fig. 5G) revealed that almost all the pathways that were downregulated upon chronic ATP synthase inhibition (Fig. S2B, C) were upregulated after the edaravone administration (Figs. 5H, S8A).

### Edaravone boosts mitochondrial fusion and biogenesis

**Fig. 6:**
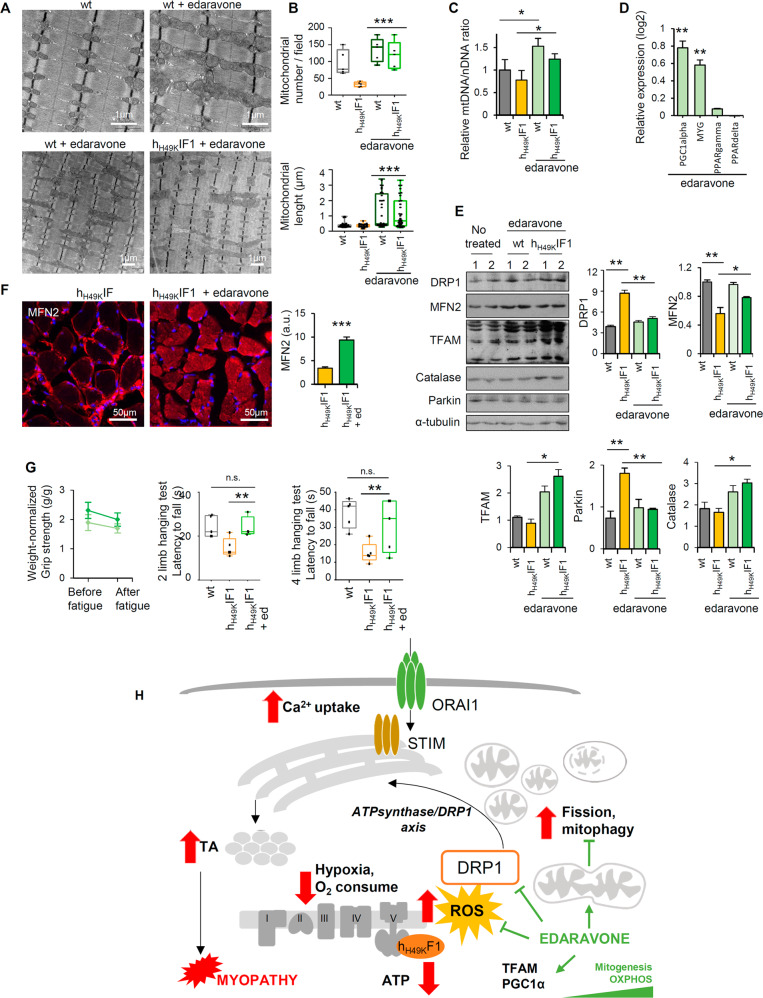
Edaravone boosts mitochondrial fusion and biogenesis, preventing TA. **A** Representative TEM images of Skm tissue in transversal slices of *soleus* from wt and h_H49K_IF1 18 months-old mice treated or not with edaravone. Images are representative of *n* = 4 mice/genotype. **B** Quantification of mitochondrial number (upper panel) and length (lower panel). Measures are taken in *n* = 4 animals/genotype, 10 images/mouse. **C** Relative mtDNA/nDNA ratio by qPCR. *n* = 3mice/genotype. **D** qPCR relative expression of proteins related to mitochondria biogenesis in edaravone-treated mice, *n* = 4/genotype. Values are expressed as h_H49K_IF1/wt ratio. Relative expression of PPARG Coactivator 1 Alpha, PGC1alpha; myoglobin, MYG, and Peroxisome Proliferator Activated Receptors (delta, and gamma) is shown. **E** WB expression of proteins related with mitochondrial dynamics, biogenesis, mitophagy, and redox system. Tubulin is shown as loading control. Two samples per condition; quantification in the right histograms. Bars are the mean ± SEM of three experiments. In histograms, the quantification of protein expression in wt and h_H49K_IF1 mice, treated or not with edaravone. *n* = at least 4 animals/genotype. **F** Immunofluorescence from transversal slices of *soleus* from h_H49K_IF1 18 months-old mice treated or not with edaravone. Red, MFN2; blue, Dapi. Histograms on the right indicate MFN2 fluorescence intensity and are the mean ± SEM of 3 experiments, *n* = 4 animals/genotype. **G** Grip-force test before and after 1 h rotarod fatigue (left) and latency to fall in 2 limb and 4 limb hanging tests (right). wt, *n* = 4; h_H49K_IF, *n* = 4; wt + edaravone, *n* = 5; h_H49K_IF1 + edaravone, *n* = 5. Bars are the mean ± SEM of *n* = 4 animals/genotype/age. **H** Schematic representation of TA formation and myopathy upon the h_H49K_IF1-mediated inhibition of the ATP synthase. Resulting OXPHOS inhibition and ROS production induce DRP1 overexpression, mitochondrial fission, and mitophagy. TA may result from the futile circle between the DRP1-mediated SOCE hyper-stimulation and the SR tubulation, presumably aimed at favouring mitochondria-SR interactions to facilitate calcium buffering. Edaravone rewires the observed phenotype by enhancing mitogenesis and mitochondrial fusion. In **B**, **G**, box plots represent 25th to 75th percentiles with the median value in the middle line, and with all data represented from minimal to maximal values. *, **, ***p < 0.05, 0.01 and 0.001 when compared to wt by ANOVA and Student’s t-test, respectively. See also Fig. S8.

In adult muscle, intermyofibrillar mitochondria are located at the I band, on both sides of the Z-line (Fig. 6A, upper panel, left, and S8B). Interestingly, in edaravone treated wt and h_H49K_IF1 mice, mitochondria were clustered in small groups or longitudinally oriented in rows between the myofibrils (Fig. 6A, S8B), similar to those observed in muscle of young mice [38]. The treatment of edaravone alone, regardless of the mouse genotype, generated larger and fused mitochondria (Fig. 6A), increasing their number and length in comparison to untreated controls (Fig. 6B). Moreover, edaravone treatment improved the mtDNA/nDNA ratio (Fig. 6C) and the PGC1α gene-expression (Fig. 6D) in mouse *soleus*. These data suggest an edaravone-dependent burst in Skm mitochondrial biogenesis and fusion. Consistently, in both genotypes a significant increase in the expression of TFAM (Fig. 6E) and MFN2 (Figs. 6E, F, S8C) was observed. Remarkably, no TA were observed in *soleus* from h_H49K_IF1 mice treated with edaravone (Figs. 6A, S8B), raising the possibility of a Skm OXPHOS threshold that may be limiting for TA setting. In agreement with literature [43], edaravone did not alter SOCE activity (Fig. S8D), and its mechanism seemed to be related with prompting mitogenesis and mitochondrial activity. This also included the normalization of DRP1, parkin and catalase expression (Fig. 6E), suggesting the reestablishment of the Skm mitophagy and redox homeostasis.

As a result, edaravone-treated h_H49K_IF1 and wt mice presented no differences in tissue architecture (Figs. 6A, S8B, E), fiber diameter (Fig. S8F) and in respiratory complex activities (Fig. S8E). Besides, they displayed similar motor performances (Fig. 6G). Although edaravone administration did not ameliorate the muscular activity of aged wt animals (Fig. S8H), in h_H49K_IF1 mice this treatment restored motor performances to the level of wt in both grip force and two- and four-limbs hanging tests (Figs. 6G, S8G, H).

Altogether, these in vivo studies identify edaravone as a promising drug in the management of TA-related muscular disorders.

## Discussion

Aimed at understanding the functional relevance of the mitochondrial dysfunction observed in TAM, the present study was designed to unveil whether the long-term ATP synthase inhibition in Skm leads to the setting of TA. Inhibiting the ATP synthase is known to produce ROS [10, 11]. ROS and dimers of the ATP synthase contribute to mitochondrial *cristae* [44] organization [19, 45], and alter the shape of the mitochondrial reticulum [31, 32]. In response to oxidative stress, the recruitment and activation of DRP1 shifts the balance toward fragmented mitochondria, and mitophagic events are favored to recycle dysfunctional organelles [33, 46]. In this regard, the chronic expression of h_H49K_IF1, altering ROS production [11] and the ATP synthase activity and dimer formation [18, 19, 47], may modify mitochondrial network in muscle, a tissue where the physiological expression of IF1 is null [18]. Remarkably, DRP1 was over-expressed in our model and mitochondria appeared smaller, fissioned, and undergoing mitophagy as an attempt to mitigate the long-term OXPHOS impairment.

The inhibition of the ATP synthase also produces the rewire of energy metabolism towards glycolysis [11] in the so-called Warburg effect [48]. In Skm, the most plastic tissue of the body, a metabolic shift may alter fiber composition [23, 24]. Accordingly, in h_H49K_IF1 mice, *soleus* presents less type IIa fibers, increasing the proportion of IIx and IIb. Besides, we do not discard the possibility of a metabolic reprogramming of oxidative fibers toward glycolysis without affecting MYHs expression. Consistently, clustering of TA formed in *soleus* upon a prolonged ATP synthesis inhibition, similar to those observed in human type-IIb-enriched muscles under pathologic conditions [2, 3]. This unusual appearance is consistent with diseases such as obesity and insulin resistance, where mitochondrial activity is impaired [49], leading to an enhanced proportion of glycolytic fibers [50, 51]. Intriguingly, metabolic disorders course with the appearance of TA in aged muscles [3]. On the contrary, when PGC-1α is overexpressed in fast muscles, it activates markers of slow muscle identity and oxidative metabolism [23]; interestingly, endurance exercise that induces PGC-1α expression [52] reduces TA in aged mice [53].

Although it is known that TA are related with SR distress and calcium overload, how TA form is unclear. The interaction among SOCE, SR, and mitochondria is complex and reciprocal [54–56]. MAMs and ERMES control fusion/fission processes, and, in turn, MFN2 and DRP1 mediate ERMES tethering at MAMs [57], finally influencing SOCE and calcium fluxes [13, 31]. Mitochondrial calcium buffering capacity, ATP, pyruvate, ROS, and mitochondrial dynamics are all elements that influence SOCE [34, 35,58]. However, the effectors of the SOCE-mitochondria interplay are still unknown. We showed that a restrained ATP synthase results in SOCE upregulation mediated by DRP1 that acts as a master regulator of the ER/SR stress signaling and morphology. It is thus reasonable to speculate that dysregulations in mitochondria-SR dynamics might be responsible for TA setting. In support of this hypothesis, TAM patients-derived myotubes show fissionated mitochondrial network [39]. One limitation is that no TA have been reported in Skm from available MFN1, MFN2, OPA-1, or DRP1 knock-out or knock-in mice. However, genetic alterations in mitochondrial dynamics have a deep impact on lifespan and all these mice died early in life even in heterozygosis [46], what prevents TA formation during aging.

An alternative hypothesis is that TA onset requires specific dysfunctions in the ATP synthase/DRP1 axis. This is consistent with the recently published ability of DRP1 to tubule the ER in a GTPase independent manner with the aim to increase mitochondrial-reticulum contact sites [59]. Supporting this idea, both hypoxia and cyanide treatment, the two conditions able to induce TA in vitro [14], increase the expression of DRP1 [60, 61]. Besides, the DRP1 inhibitor mDIVI prevents TA formation during hypoxia in our model. Therefore, TA may be the result of the ATP synthase-mediated DRP1 over-expression, aimed at favouring mitochondria replacement and mitochondria-SR interactions [59], in a futile attempt to facilitate calcium detoxification upon prolonged OXPHOS inhibition (Fig. 6H).

Based on this, boosting Skm mitochondrial activity and reducing fission may be a viable therapeutic approach for TA-related diseases. Accordingly, the in vivo administration of the mitochondrial enhancer [18] edaravone [42] in mice prevents the setting of TA. Consistent with beneficial effects of mitochondrial fusion agonizts in other neuromuscular diseases [62], we show that the mechanism of edaravone is related with a burst in mitochondrial biogenesis and fusion. Because Skm mitochondrial dysfunction is a common trait in TAM patients, edaravone represents a promising drug in the management of TAM and TA-related disorders whose current treatment is limited to supportive care of the patients.

## Materials and methods

### Reagents

Edaravone was used at 2 μM (in vitro) or injected intraperitoneally (i.p.) at 3 mg/kg (in vivo). A comprehensive list of reagents, primers, and antibodies utilized is detailed in Table S1.

### Ethical considerations

All human and animal studies were performed following EU ethical and ARRIVE guidelines. Animal procedures have the approval of the Institutional Review Board (UAM University and Madrid Community Ethical Committees, Spain; CEI-24-571, PROEX 183/17).

### Human Skm biopsies

Human samples were provided by the “Biobank of Cells, tissues, and DNA from patients with neuromuscular diseases”, and the “Biobank of skeletal muscle, peripheral nerve, DNA and cell lines”, members of the Telethon Network of Genetic Biobanks and of the EuroBioBank network.

### Animal studies

B6;C3-Tg(ACTA1-rtTA,tetO-cre)102MonK/J mice (h_H49K_IF1|T) were purchased from The Jackson Laboratories. The Tet-on double transgenic h_H49K_IF1|T/H (h_H49K_IF1) mouse [18] was obtained by breeding h_H49K_IF1|T with the h_H49K_IF1|H mouse [22], which integrates in its genome the h_H49K_IF1-TRE construct under a tetracycline-regulated promoter. Mice were maintained on the (C57BL/6x C3H)F2 background. Administration of 2 mg/ml doxycycline in the drinking water for at least 2 weeks was used to turn on the Skm expression of h_H49K_IF1 protein. TAM and TA were never observed in women [3, 5] or female inbred mice [4]. For this reason, in this study we used exclusively male mice. All experiments were performed on age-matched littermate wt and h_H49K_IF1|T/H mice (6 month-old or 18 month-old mice).

In order to minimize the number of animals we used power analysis to calculate the minimum sample size using the free software DOEUMH (https://samplesizeumh.shinyapps.io/DOEUMH) based on the TrialSize library of the R program (R Core Team). We selected the procedure KMeans – ANOVA, fixing the significance to 0.05, power to 0.08, and a drop-out of 5%. We took into consideration differences between averages of about 1.5–2 fold. Minimum number of mice/group: 5–6 mice/group.

Mouse motor functions were evaluated by (i) Grip strength test; (ii) Rotarod; (iii) Tail Suspension Test (TST), and (iv) 2- and 4-Limbs Hanging Tests as previously reported [18]. All tests were performed in a blinded fashion. Randomization was assessed by equally distributing experimental groups across multiple cages and balancing the location of the mouse cages on the racks.

#### Grip force/strength test

The grip force test was used to measure the maximum strength that could be performed by a mouse with its forelimbs by taking advantage of the animal’s tendency to grasp to surfaces. One mouse at a time was let to grasp the metallic bars and then was gently pulled away until its grasp is broken. The tests were run by the same investigator who placed her elbow on top of the bench, and at the same distance from the apparatus, to exert the minimum force required to break the grasp. The pulling was performed sufficiently slowly to permit the mouse to build up a resistance against it. Force (*g*) was recorded by a grip force meter (Harvard Apparatus). The test was repeated five times per mouse, with at least 1 min elapsing between each determination.

#### Rotarod test

The rotarod test was performed in 3 days. During first and second days, mice were trained, and the third day the test was performed. For all tests, a soft padded surface was placed at the base of the apparatus to cushion any mice that fall off.

Day 1 was used to train the mice in one session of 2 min, walking at 4 rpm. Mice that fell off after completing 2 min walking within 10 min were discarded from the experiment.

Day 2- training consists of: (a) 15 min on the rod (run speed: 13 rpm) per animal. When mice fell off, they were put back on the rod. (b) 30 min rest. (c) 15 min on the rod (run speed ramping from 13 to 20 rpm in 180 s) per animal.

Day 3- test: start speed, 4 rpm; acceleration, 8 rpm/min; maximum speed, 20 rpm. Animals were let on the rod until they fell out. Their latency was measured. Mice that fell off 4 times within 60 s were discarded from the experiment.

#### Fatigue task

Includes both grip strength and rotarod. Training for rotarod was performed as described. In the fatigue test day, mice force was measured 5 times by grip strength before placing them on the rod, followed by 1.5 h in the rod at 20 rpm constant speed. Afterwards, mice force was measured again for 5 times. If a mouse fell off the rod four times within 60 s, it was termed fatigued and the rotarod task stopped.

#### Tail suspension test (TST)

The TST is used to recognize and evaluate dyskinesia and abnormal movements (hindlimb clasping) of mice when subjected to vertical suspension of the tail for 30 s. Their responses were scored as follows: “0” when the hindlimbs were completely extended (normal wild-type display), “1” when one or both hindlimbs were intermittently extended and bent, and “2” when both hindlimbs were completely bent and folded into the abdomen. Data was then represented in % of the wt.

#### Hanging test: two limbs

The forelimbs muscle strength was measured by monitoring the ability of mice to exhibit sustained limb tension to oppose their weigh. Mice were placed in a 2 mm thick metal bar at 35 cm of a padded surface and time until falling was recorded. The test ended after a hanging time of 2 min was achieved or otherwise after three sessions. Longest hanging time (s) and minimal holding impulse (body mass × hang time) were calculated.

#### Hanging test: four limbs

The four limbs muscle strength was measured by monitoring the ability of mice to exhibit sustained limb tension to oppose their weight. Mice were placed in a wire grid at 35 cm of a padded surface and time until falling was recorded. The test ended after a hanging time of 2 min was achieved or otherwise after three sessions. Longest hanging time (s) and minimal holding impulse (body mass × hang time) were calculated.

#### Soleus and gastrocnemius isolation

After sacrifice, *soleus* and *gastrocnemius* muscles from wt and h_H49K_IF1 mice were dissected from hindlimbs after hair, skin, and surrounding fascia removal. Achilles tendon was cut as close as possible to the knee and muscles separated with no white adipose tissue (WAT) deposits.

### Cell culture and transfection

Primary cultures of myoblasts derived from wt and h_H49K_IF1 mice or C_2_C_12_ cells were cultured in growing media (DMEM 10% FBS, 1 mM glutamine, and amino acids) at 37 °C and 10% CO_2_. When needed, myoblasts at 80% confluence were differentiated to myocytes by 48–72 h in D-media (DMEM 2% FBS, 1 mM glutamine and amino acids, 100 nM insulin).

At ~70% confluence, 3 × 10^5^ myoblasts were transfected with CRL or h_H49K_IF1 plasmid (pCMV-SPORT6- h_H49K_IF1 or pCMV-SPORT6-control, [49]) using lipofectamine 3000 transfection reagent (Invitrogen) and following the manufacturer’s instructions. Experiments were performed 24 h post transfection.

### Stable DRP1-ko myoblasts

DRP1–/– myoblast were obtained using CRISPR-Cas9 technology as described in [63]. Single-guide RNAs (sgRNAs) were obtained by an online CRISPR Design Tool (http://www.rgenome.net/cas-designer/), targeting the exon 2 of DRP1 (sequence: GGCAGGGACCTTCTTCCCAG). sgRNAs were cloned into pSpCas9(BB)-2A-GFP plasmid and C_2_C_12_ cells were transfected with this plasmid and incubated for 48 h. After, cells were sorted, and GFP-expressing cells were selected and cultured in previously described conditions. The DRP1 CRISPR-Cas9-mediated gene knock-out was checked by WB.

### Calcium uptake measurements in living cells

Wt and DRP1–/– myoblast expressing or not h_H49K_IF1 were used.

#### SOCE activity

Cells were seeded on coverslips and loaded with 2 mM calcium, 5 μM Fura-2AM (Invitrogen), 3 mg/ml probenecid (Sigma) and 50 μM pluronic acid F.127 (Invitrogen) in PSS (145 mM NaCl, 5 mM KCl, 1 mM MgCl_2_, 5 mM Hepes, 10 mM glucose, pH 7.4) for 1 h at 37 °C. Cells were washed 5 min in PSS with 2 mM EGTA to remove calcium and experiments assessed in PSS + 2 mM EGTA + 1 µM thapsigargin. Coverslips were mounted on a microscope stage equipped with a 40× objective as described previously [35] and Fura-2 fluorescence was imaged ratiometrically using alternate excitation at 340 and 380 nm, and a 510 nm emission filter with a Neofluar40×/0.75 objective in an Axiovert 75 M microscope (Zeiss). At time = 2 min a single bolus of 2 mM Calcium was added to observe the SOCE-dependent calcium uptake. For single-cell analysis of [Ca^2+^]i the ratio of fluorescence intensity at 340 nm (F340) and 380 nm (F380) (F340/F380) was obtained. Image acquisition was performed with the Aquacosmos 2.5 software (Hamamatsu) and data analysis was done with Aquacosmos 2.5, Excel, and GraphPad Prism 7.0 softwares.

#### Membrane and mitochondrial calcium signaling determination (FRET)

Cells were seeded on coverslips and transfected with Lipofectamine 3000 following the manufacturer’s instructions with pcDNA-lynD3cpv (directed to the plasmatic membrane) or pcDNA-4mtD3cpv (directed to the mitochondria) [54]. Cells were excited 100 ms at 436/20 mm, and the emission was detected through a dichroic dual pass CFP-YFP (changing 440/500 nm for CFP and 510/600 nm for cpV). Images were registered every 5 s with a PCO edge 4.2 bi sCMOS camera in a Axiovert 200 M inverted microscope (Zeiss) equipped with an 40×/1.4 oil Plan-Apochromat Ph3 objective. The cpV/CFP ratio was determined with a ROI for each transfected cell and the fluorescent signaling was analyzed using Metamorph 7.1 r2 Software (Universal Imaging) and Fiji (NIH) [54].

#### Calcium retention capacity of isolated mitochondria (CRCs)

The CRC of isolated mitochondria was measured with the Ca^2+^-sensitive fluorescent probe Calcium-Green 5N (0.1 μM, excitation 506 nm, emission 532 nm) in MSK medium (75 mM manitol, 25 mM saccharose, 5 mM KH2PO4, 20 mM TrisHCl, 100 mM KCl, 0.1% BSA; pH 7.4) using a BMG labtech FLUOstar OTIMA plate reader. All experiments were carried out at 30 °C in the presence of 1 mM MgCl_2_, respiratory substrates (5 mM succinate + 2 μM rotenone) and in the presence or absence of 50 µM ADP. 0.3 mg/ml of mitochondria were challenged with subsequent 10 µM CaCl_2_ additions after reaching a baseline, and Ca^2+^ uptake into mitochondria was measured as a decrease in fluorescence.

### Oxygen consumption rate (OCR)

Myocytes OCR was determined in an XF24-Extracellular Flux Analyzer with the XFe24-Flux Pack following the manufacturer’s protocol. Glucose (4.5 mM) was used as main substrate. The concentration and order of injected substances was 5 μM oligomycin, 0.25 mM DNP, 1 μM rotenone, and 1 μM antimycin A.

### TA generation by Hypoxia (ex vivo muscle cultures)

*Gastrocnemius* muscles were dissected from hind limbs after hair, skin, and surrounding fascia removal. Achilles tendon was cut as close as possible to the knee and muscles separated without WAT deposits [64]. Rapidly after dissection, muscles were placed in p24 plates with 500 µl of complete DMEM (previously maintained in hypoxia for 24 h) and cultured in hypoxia (0.5% O_2_). When indicated, 5 µm mDIVI was added to the media. After 6 h in the hypoxic chamber, muscles were washed, fixed in 4% paraformaldehyde and 2% glutaraldehyde in 0.1 M phosphate buffer and processed for TEM.

### On-slice mitochondrial complex activities

*Soleus* frozen slices from wt and h_H49K_IF1 mice were incubated with the following solutions for assessing the specific ETC complex activities. Complex I: 5 mM Tris HCl, pH 7.4, 1 NTB tablet (5 mg; NitroBlue Tetrazolium Tablet), and 10 mg/ml NADH. Complex IV: 50 mM phosphate buffer (NaH_2_PO_4_ + NaHPO_4_, pH 7.2), 5 mg DAB, and 5 mM reduced cytochrome C. Stop solution: 50:50 MeOH/PBS.

### Mitochondrial ROS

The mitochondrial production of superoxide (mitoROS) in myocytes was monitored by flow cytometry using 5 μM MitoSox [11]. Cells were analyzed in a BD-FACScan. MitoQ (20 nM) was used as positive antioxidant control. Data were analyzed in FlowJo software v10.6.2.

### TAGs and glycerol

For FFA and free glycerol determination, 40 mg of Skm tissue were homogenized in 1 ml of 2-isopropanol in a TissueLyser (Qiagen). Five microliters of Skm extracts were used for TAGs quantification using the Glycerol Quantification Kit (Sigma-Aldrich) and a FLUOstar-Omega spectrophotometer (BMG Labtech). All results were adjusted for exact protein content.

### TEM microscopy

Sample preparation was performed by the Electron Microscopy Facility at the CBMSO, UAM University, Spain. *Soleus* muscles were fixed with 4% paraformaldehyde and 2% glutaraldehyde in 0.1 M phosphate buffer and treated with 1% osmium tetroxide at 4 °C for 1 h. Then, they were dehydrated with EtOH and embedded in TAAB812 epoxy resin. Ultrathin 80 nm sections of the embedded tissue were obtained using an ultramicrotome Ultracult E (Leica) and mounted on carbon-coated copper 75-mesh grids. The sections were stained with uranyl acetate and lead citrate and examined at 80 kv in a JEOL JEM 1010 electron microscope. Images were recorded with a TemCam F416 (4k × 4 K) digital camera from TVIPS. Sarcomere and mitochondrial measurements were performed using ImageJ 1.52r software.

### Immunofluorescence, confocal and optic microscopy

*Soleus* muscles were fresh frozen (OCT) or PFA-fixed, sliced, and histologically prepared (and stained with hematoxylin/eosin) by the Histology Facility at CNB-CSIC, UAM University, Spain. For freshly frozen preparations, nitrogen-chilled methylbutane was used as OCT freezing medium and slices freshly cut by cryostat. The muscle PFA-fixed sections were subjected to 20 min heat-induced epitope retrieval with sodium citrate buffer (10 mM sodium citrate, 0.05% Tween 20, pH 6.0). Dyes were incubated in 3% BSA, 5% horse serum in PBS with 0.1% Triton-X-100 (antibody solution). Stainings: laminin (1:1000); MFN2 (1:500); DAPI (1:1000) for nuclei. MYHs specific markers: type I fibers: MYH7, antibody clone: BA-F8 (1:50); type IIa fibers: MYH2, antibody clone: SC-71 (1:100); type IIb fibers: MYH4, antibody clone: DF-F3(1:100); type IIx fibers: antibody clone: BF-35, all but type IIx fibers (1:50). Images were acquired on a Leica DMRE light microscope or by confocal microscopy using a Bio-Rad Radiance 2000 Zeiss Axiovert S100TV.

### Real-time PCR

DNA or RNA was purified from 100 mg of *soleus* and *gastrocnemius* muscles from h_H49K_IF1 and wt mice following a standard TRIzol/chloroform method. Purified RNA (1 μg) was retrotranscribed into cDNA using the High-Capacity cDNA Reverse Transcription Kit. Real-time PCR was performed using the Fast SYBR Master Mix and ABI Prism 7900HT sequence detection system at the Genomics and Massive Sequencing Facility (CBMSO–UAM). Actin and Glyceraldehyde phosphate dehydrogenase (GAPDH) were selected as housekeeping genes to normalize the mRNA levels. All primer sequences are included in Table S1.

The mtDNA/nDNA ratio was performed using Atp5g, M2B and SDHA as nuclear genes and ND4 as mitochondrial gene, following the equation:

$$2^ \ast \left( {2\Delta {{{\mathrm{Ct}}}}} \right),\;{{{\mathrm{where}}}}\;\Delta {{{\mathrm{Ct}}}} = {{{\mathrm{Ct}}}}\left( {{{{\mathrm{mtDNA}}}}\;{{{\mathrm{gene}}}}} \right) - {{{\mathrm{Ct}}}}\left( {{{{\mathrm{nDNA}}}}\;{{{\mathrm{gene}}}}} \right).$$

Standard curves with serial dilutions of pooled cDNA were used to assess the amplification efficiency of the primers and to establish the dynamic range of cDNA concentration for amplification. SDS 2.4 software was used for data collection, and the relative expression of the mRNAs was determined with the comparative ΔΔCt method.

### Protein extraction and western blot analysis

*Soleus* or *gastrocnemius* muscles were extracted in lysis buffer containing 25 mM HEPES, 2.5 mM EDTA, 1% Triton X-100 supplemented with protease and phosphatase inhibitor cocktails. Lysates were freezed/thawed three times and clarified. The resulting supernatants were fractionated on SDS-PAGE and transferred onto nitrocellulose membranes for immunoblot analysis. Protein concentrations were determined using Bradford reagent (Bio-Rad protein assay). The primary antibodies used are detailed in Table S1. Peroxidase conjugated anti-mouse or anti-rabbit IgGs (Promega, 1:3000) were diluted in 5% non-fat dried milk in Tris Buffered Saline (TBS) with 1% Tween 20 and used as secondary antibodies. The Novex® ECL (Thermo Fisher Scientific) system was used to visualize the bands. WB quantifications are reported in Fig. S9.

### High-precision antibody microarray (iTWO-300 RPPA)

Proteins were extracted from ~10 mg human Skm biopsies using a BEAD MILL 24 homogenizer and beads in a ratio of 1:8 w/v with TPER buffer supplemented with proteases and phosphatases inhibitor cocktails. iTWO-300 protein array was performed as described [20]. In detail, lysates were freezed/thawed 3 times and clarified by centrifugation at 13,000 × *g* for 30 min at 4 °C. Protein concentrations were quantified by Bradford method, and lysates diluted to 2 µg/µl in TPER buffer (sample stock solution), what allowed the array machine to print 40 µL/sample in 1:4 TPER/PBS printing buffer on array slides, reaching a final concentration of 0.5 µg protein/sample.

Slide pattern of printing was designed by loading the slide map (SOURCE) and sample information Excel (CSV) in the array system using the InDot software. Sample printing was assessed by Piezo-Driven Micro-Dispenser 30–150pl (PDMD) at controlled 52% humidity conditions. Standard curves of BSA (stained with Fast Green FCF, negative control), IgG, C_2_C_12_, and HCT116 cell lines were used as controls.

#### Antibody incubation and revealing

After printing, slides were kept at 4 °C for 24 h, dried, and blocked (except for FCF wells) in Super G blocking buffer for 2 h at RT. Primary and fluorophore-conjugated secondary antibodies were incubated O/N at 4 °C and 1 h in obscurity at RT, respectively. FCF wells were incubated with 1× PBS (5 min); 10% MetOH + 7% acetic acid (5 min); 0.0001% FCF in 30% MetOH + 7% acetic acid (35 secs); MetOH 10% + 7% acetic acid (3 × 20 s); 10% EtOH + 7% acetic acid (3 × 20 s) and EtOH + 7% acetic acid (30 min). After secondary antibody incubation, slides were washed (except for FCF wells) with 1× PBS + Tween (3 × 10 min); 1× PBS (3 × 2 min), and mQ H_2_O (2 × 30 s). Slides were dried using a vacuum bomb and fluorescence signal detected in a Typhoon 9410 apparatus (560 nm long pass for Alexa 647 and Red 633 laser).

### Quantitative proteomics (TMT)

TMT sixplex Isobaric Mass Tagging analysis was carried out in the CBMSO Protein Chemistry Facility (ProteoRed, PRB3-ISCIII, and UAM University, Spain).

#### In-gel digestion (stacking gel)

Protein extracts were fractionated in a SDS-PAGE gel (0.75 mm-thick, 4% stacking, and 10% resolving). The run was stopped as soon as the front entered 3 mm into the resolving gel, so that the whole proteome became concentrated in the stacking/resolving gel interface. The unseparated protein bands were visualized by Coomassie staining, excised, cut into cubes (2 × 2 mm), and placed in 0.5 ml microcentrifuge tubes. The gel pieces were destained in acetonitrile:water (ACN:H_2_O, 1:1), reduced and alkylated (disulfide bonds from cysteinyl residues were reduced with 10 mM DTT for 1 h at 56 °C, and then thiol groups were alkylated with 10 mM iodoacetamide for 30 min at room temperature in darkness) and digested in situ with sequencing grade trypsin (Promega, Madison, WI). The gel pieces were shrunk by removing all liquid using sufficient ACN. Acetonitrile was pipetted out and the gel pieces were dried in a speedvac. The dried gel pieces were re-swollen in 100 mM Tris-HCl pH 8, 10 mM CaCl_2_ with 60 ng/µL trypsin at 5:1 protein:enzyme (w/w) ratio. The tubes were kept in ice for 2 h and incubated at 37 °C for 12 h. Digestion was stopped by the addition of 1% TFA. Whole supernatants were dried down and then desalted onto OMIX Pipette tips C18 (Agilent Technologies) until the mass spectrometric analysis.

### TMT labeling and high pH fractionation

#### TMT

The resultant peptide mixture from desalted proteins tryptic digest (60 µg) was labeled using chemicals from the TMT sixplex Isobaric Mass Tagging Kit (Thermo Fisher Scientific, MA, USA) as described by the manufacturer. Briefly, peptides were dissolved in 50 μL of 100 mM triethylammonium bicarbonate (TEAB), adjusted to pH 8. For labeling, each TMT reagent was dissolved in 41 μL of ACN and added to the respective peptide mixture and then incubated at room temperature for 1 h. Labeling was stopped by the addition of 8 μL 5% hidroxilamine. Whole supernatants were dried down and the four samples were mixed to obtain the “4plex-labeled mixture”. The mixture was analyzed by RP-LC-MS/MS to check the efficiency of the labeling.

#### Fractionation

The sample was then fractionated using the Pierce High pH Reversed-Phase Peptide Fractionation Kit (Thermo Fisher Scientific, MA, USA) with minor modifications. Sample were re-swollen in 0.1% TFA and then, loaded onto an equilibrated, high-pH, reversed-phase fractionation spin column. A step gradient of increasing acetonitrile concentrations (5–80%) in a volatile high-pH (Triethylamine (0.1%)) is then applied to the columns to elute bound peptides into nine different fractions collected by centrifugation. The fractions obtained from high-pH, reversed-phase 6plex-labeled mixture were dried and stored until analysis by mass spectrometry for quantification.

#### Quantitative analysis by reverse phase-liquid chromatography rp-lc-ms/ms

The fractions were resuspended in 10 µL of 0.1% formic acid and analyzed by RP-LC-MS/MS in an Easy-nLC II system coupled to an ion trap LTQ-Orbitrap-Velos-Pro hybrid mass spectrometer (Thermo Scientific). The peptides were concentrated (on-line) by reverse phase chromatography using a 0.1 mm × 20 mm C18 RP precolumn (Thermo Scientific), and then separated using a 0.075 mm × 250 mm C18 RP column (Thermo Scientific) operating at 0.3 μl/min. Peptides were eluted using a 90 min dual gradient. The gradient profile was set as follows: 5–25% solvent B for 68 min, 25–40% solvent B for 22 min, 40–100% solvent B for 2 min, and 100% solvent B for 18 min (Solvent A: 0.1% formic acid in water, solvent B: 0.1% formic acid, 80% ACN in water). ESI ionization was done using a Nano-bore emitters Stainless Steel ID 30 μm (Proxeon) interface at 2.1 kV spray voltage with S-Lens of 60%. The instrument method consisted of a data-dependent top-20 experiment with an Orbitrap MS1 scan at a resolution (m/Δm) of 30,000 followed by either twenty high energy collision dissociation (HCD) MS/MS mass-analyzed in the Orbitrap at 7500 (Δ*m*/*m*) resolution. MS2 experiments were performed using HCD to generate high resolution and high mass accuracy MS2 spectra. The minimum MS signal for triggering MS/MS was set to 500. The lock mass option was enabled for both MS and MS/MS mode and the polydimethylcyclosiloxane ions (protonated (Si(CH3)2 O))6; m/z 445.120025) were used for internal recalibration of the mass spectra. Peptides were detected in survey scans from 400 to 1600 amu (1 μscan) using an isolation width of 1.3 μ (in mass-to-charge ratio units), normalized collision energy of 40% for HCD fragmentation, and dynamic exclusion applied during 60 s periods. Charge-state screening was enabled to reject unassigned and singly charged protonated ions.

#### Quantitative data analysis

Peptide identification from raw data (a single search was performed with all nine rows from the fractionation) was carried out using PEAKS Studio Xpro search engine (Bioinformatics Solutions Inc., Waterloo, Ontario, Canada). Database search was performed against uniprot-mus-musculus.fasta (55466 entries; UniProt release 08/2020) (decoy-fusion database). The following constraints were used for the searches: tryptic cleavage after Arg and Lys (semispecific), up to two missed cleavage sites, and tolerances of 20 ppm for precursor ions and 0.05 Da for MS/MS fragment ions, and the searches were performed allowing optional Met oxidation and Cys carbamidomethylation and fixed TMT 6plex reagent labeling at the N-terminus and lysine residues. False discovery rates (FDR) for peptide spectrum matches (PSM) was limited to 0.01. Only those proteins with at least two distinct peptides and at least one unique peptide being discovered from LC/MS/MS analyses were considered reliably identified and sent to be quantified.

Quantitation of TMT labeled peptides was performed with PEAKS Studio Xpro search engine, selected “Reporter Ion Quantification iTRAQ/TMT” under the “Quantifications” options. We use Auto normalization mode that calculates a global ratio from the total intensity of all labels in all quantifiable peptides. The -10LgP, Quality [13], and Reporter Ion Intensity (1e4) were used for Spectrum filter and Significance (20, PEAKSQ method) was used for peptide and protein abundance calculation. For the protein quantification, we consider protein groups for peptide uniqueness and use only unique peptides for protein quantification.

After normalization and filtering steps, proteomic data were analyzed by Gene Set Enrichment Analysis (GSEA v4.1.0, http://www.gsea-msigdb.org/gsea/index.jsp) and visualized by Cytoscape v3.6.1 free software.

GENEPIX 5.1 and InDot software were used for iTWO-300 RPPA initial data charging and densitometric analysis, respectively. ImageJ 1.52r software was used for quantification and image analysis.

### Statistical analyses

Statistical analyses were performed using a two-tailed Student’s t test. ANOVA and the Tukey’s post hoc test were used for multiple comparisons, employing SPSS 17.0 and GraphPad Prism7 software packages. When multiple t-tests were performed, Bonferroni correction was applied to avoid multiple comparison errors. The results shown are the means ± SEM. A *p* < 0.05 was considered statistically significant. The *n* used in each statistical test is indicated in the Figure Legends.

## Supplementary information

Supplemental Figures and Table

AJ-checklist

Uncropped WB

Agreement with final author list

## Data Availability

Data are available from the corresponding author upon reasonable request. Skm TMT proteomics are available via ProteomeXchange with identifiers PXD026771 and PXD026722.

## Materials availability

Materials are available from the corresponding author upon material transfer agreement.
