# Supplementary material for: Chronic inhibition of the mitochondrial ATP synthase in skeletal muscle triggers sarcoplasmic reticulum distress and tubular aggregates
Source: Cell Death Dis. 2022 Jun 22;13(6):561. doi: 10.1038/s41419-022-05016-z (PMC9217934; doi:10.1038/s41419-022-05016-z)
Supplement: Supplementary file 1 — Supplemental Figures and Table [file 41419_2022_5016_MOESM1_ESM.pdf]

Fig S1

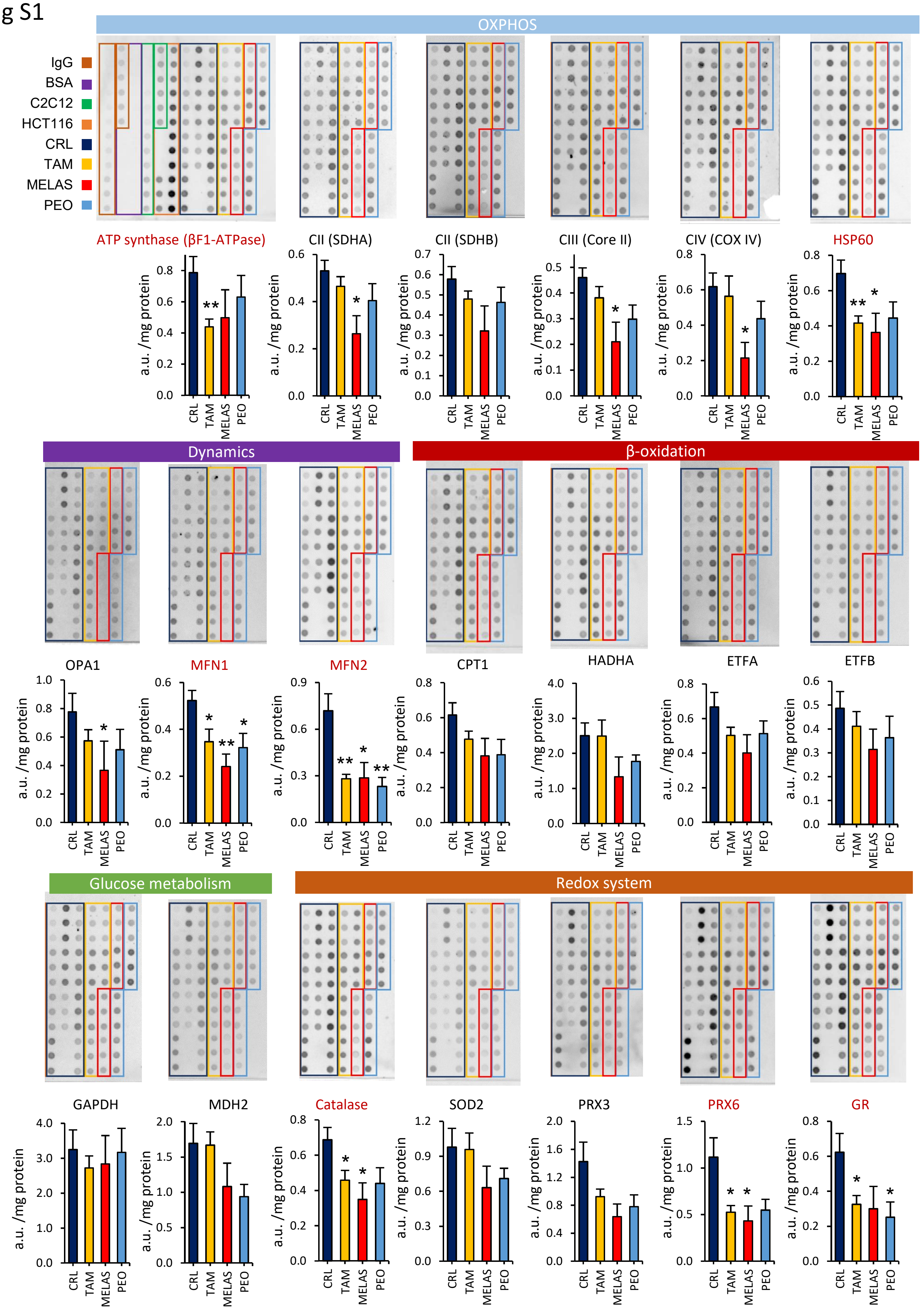

OXPHOS
IgG
BSA
C2C12
HCT116
CRL
TAM
MELAS
PEO
ATP synthase (βF1-ATPase)
CII (SDHA)
CII (SDHB)
CIII (Core II)
CIV (COX IV)
HSP60
Dynamics
β-oxidation
OPA1
MFN1
MFN2
CPT1
HADHA
ETFA
ETFB
Glucose metabolism
Redox system
GAPDH
MDH2
Catalase
SOD2
PRX3
PRX6
GR
a.u. /mg protein

Fig S2

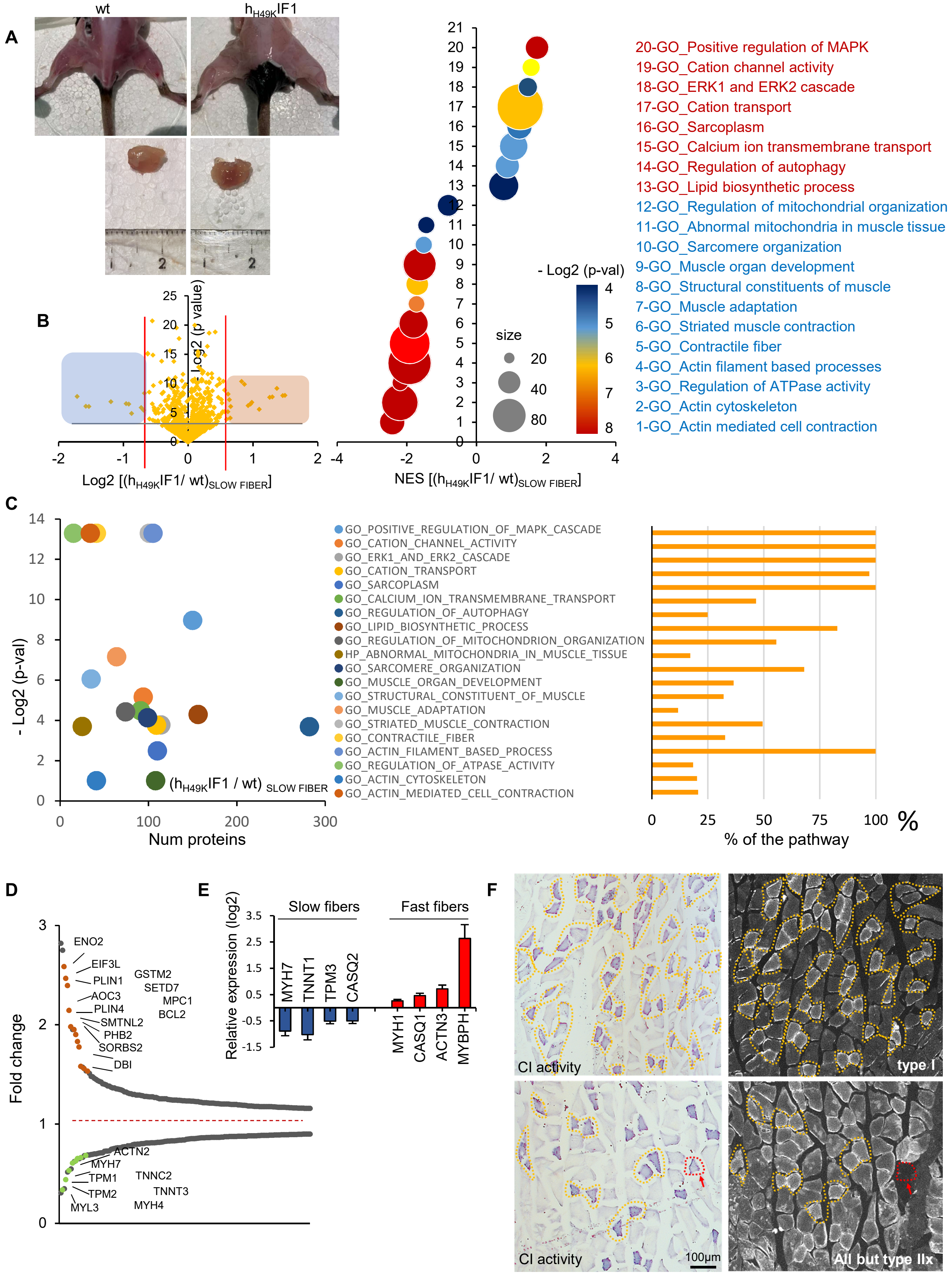

A
wt
hH49KIF1
B
Log2 [(hH49KIF1/ wt)SLOW FIBER]
- Log2 (p value)
NES [(hH49KIF1/ wt)SLOW FIBER]
- Log2 (p-val)
size
20-GO_Positive regulation of MAPK
19-GO_Cation channel activity
18-GO_ERK1 and ERK2 cascade
17-GO_Cation transport
16-GO_Sarcoplasm
15-GO_Calcium ion transmembrane transport
14-GO_Regulation of autophagy
13-GO_Lipid biosynthetic process
12-GO_Regulation of mitochondrial organization
11-GO_Abnormal mitochondria in muscle tissue
10-GO_Sarcomere organization
9-GO_Muscle organ development
8-GO_Structural constituents of muscle
7-GO_Muscle adaptation
6-GO_Striated muscle contraction
5-GO_Contractile fiber
4-GO_Actin filament based processes
3-GO_Regulation of ATPase activity
2-GO_Actin cytoskeleton
1-GO_Actin mediated cell contraction
C
- Log2 (p-val)
Num proteins
(hH49KIF1 / wt) SLOW FIBER
GO_POSITIVE_REGULATION_OF_MAPK_CASCADE
GO_CATION_CHANNEL_ACTIVITY
GO_ERK1_AND_ERK2_CASCADE
GO_CATION_TRANSPORT
GO_SARCOPLASM
GO_CALCIUM_ION_TRANSMEMBRANE_TRANSPORT
GO_REGULATION_OF_AUTOPHAGY
GO_LIPID_BIOSYNTHETIC_PROCESS
GO_REGULATION_OF_MITOCHONDRION_ORGANIZATION
HP_ABNORMAL_MITOCHONDRIA_IN_MUSCLE_TISSUE
GO_SARCOMERE_ORGANIZATION
GO_MUSCLE_ORGAN_DEVELOPMENT
GO_STRUCTURAL_CONSTITUENT_OF_MUSCLE
GO_MUSCLE_ADAPTATION
GO_STRIATED_MUSCLE_CONTRACTION
GO_CONTRACTILE_FIBER
GO_ACTIN_FILAMENT_BASED_PROCESS
GO_REGULATION_OF_ATPASE_ACTIVITY
GO_ACTIN_CYTOSKELETON
GO_ACTIN_MEDIATED_CELL_CONTRACTION
% of the pathway
D
Fold change
ENO2
EIF3L
PLIN1
AOC3
PLIN4
SMTNL2
PHB2
SORBS2
DBI
GSTM2
SETD7
MPC1
BCL2
ACTN2
MYH7
TPM1
TPM2
MYL3
TNNC2
TNNT3
MYH4
E
Relative expression (log2)
Slow fibers
Fast fibers
MYH7
TNNT1
TPM3
CASQ2
MYH1
CASQ1
ACTN3
MYBPH
F
CI activity
type I
CI activity
100μm
All but type IIx

Fig S3

Soleus / Gastrocnemius ratio

Glycolysis

Gene	wt	h_{H49K}IF1
PYGB	0.94	1
PYGL	0.95	0.89
PYGM	0.7	0.63
AGL	0.7	0.76
PGM1	0.6	0.54
PGM5	0.7	0.76
HK2	1.2	1.19
GPI	0.8	1.3
PFKM	0.7	0.78
FBP2	0.7	0.76
ALDOA	0.92	0.8
ALDOART2	0.85	0.84
TALDO1	1.19	1.21
BPGM	0.7	1.07
TPI1	0.7	0.81
PGK1	0.7	0.82
PGAM1	1.44	1.13
PGAM2	0.81	0.86
ENO2	0.7	1.19
PKM	0.7	0.66
LDHA	0.66	0.56
GPD2	0.66	0.75
ME1	0.61	0.73

Nucleotide biosynthesis

Gene	wt	h_{H49K}IF1
AK3	1.54	1.42
NNT	0.96	1.34
AMPD1	0.69	0.93
CKM	0.68	0.59
PRPS1	0.45	0.59

TCA cycle

Gene	wt	h_{H49K}IF1
IDH2	3.37	3.37
PDK4	1.96	1.58
MDH1	1.7	1.74
GOT2	1.69	1.75
CS	1.63	1.37
GPT	1.63	1.24
PDK2	1.62	1.34
SDHA	1.54	1.51
ACO2	1.5	1.38
FH	1.49	2.15
MDH2	1.49	1.44
PDHB	1.46	1.29
DLST	1.46	1.46
IDH3A	1.45	1.4
SDHB	1.44	1.39
SUCLA2	1.41	1.38
PDHA1	1.37	1.28
DLD	1.37	1.19
IDH3B	1.36	1.13
IDH3G	1.35	1.25
PDHX	1.33	1.39
MPC2	1.3	1.51
IDH1	1.3	1.01
SUCLG1	1.3	1.38
SUCLG2	1.3	1.16
DLAT	1.19	1.24

Redox system

Gene	wt	h_{H49K}IF1
PRDX5	1.97	1.51
CAT	1.9	1.4
SOD2	1.62	1.58
PRDX3	1.47	1.28
PRDX2	1.44	1.36
PRDX6	1.41	1.14
MSRA	1.37	1.25

Soleus / Gastrocnemius ratio

OXPHOS – CI

Gene	wt	h_{H49K}IF1
NDUFB10	1.82	1.61
NDUFB5	1.66	1.57
NDUFS3	1.65	1.75
NDUFV1	1.62	1.4
NDUFA6	1.57	1.6
NDUFA9	1.57	1.42
NDUFS2	1.57	1.27
NDUFA3	1.55	1.39
NDUFB4	1.54	1.55
NDUFA7	1.54	1.97
NDUFB8	1.54	1.39
NDUFA12	1.53	1.65
NDUFV2	1.53	1.52
NDUFC2	1.5	1.49
NDUFA10	1.49	1.42
NDUFS1	1.47	1.48
NDUFB9	1.46	0.59
NDUFA2	1.43	1.63
NDUFB6	1.43	1.4
NDUFS7	1.42	1.39
NDUFA5	1.42	1.47
NDUFS4	1.37	1.44
	1.21	1.26

OXPHOS – CII

Gene	wt	h_{H49K}IF1
SDHA	1.54	1.51
SDHB	1.44	1.39

OXPHOS – CIII

Gene	wt	h_{H49K}IF1
CYCS	1.82	1.71
UQCRB	1.7	1.39
MTCO2	1.54	1.78
CYC1	1.43	1.27
UQCRQ	1.4	1.28
UQCRC1	1.39	1.45
UQCRC2	1.36	1.2
UQCRFS1	1.32	1.42

OXPHOS – CIV

Gene	wt	h_{H49K}IF1
COX5B	2.3	2.08
COX5A	2.22	1.53
COX4I1	2.21	2.36
NDUFA4	1.57	2.14
COX6C	1.53	1.59
COX7A1	1.42	1.89
COX6B1	1.3	1.21

OXPHOS – CV

Gene	wt	h_{H49K}IF1
ATP5F1D	2	1.64
ATP5PD	1.91	1.36
ATP5F1B	1.67	1.51
ATP5F1C	1.63	1.35
ATP5F1E	1.61	2.3
ATP5ME	1.58	1.71
ATP5F1A	1.57	2
ATP5PB	1.48	1.47
ATP5PO	1.38	1.61
ATP5MG	1.05	1.2

CoQ synthesis

Gene	wt	h_{H49K}IF1
COQ8A	2.21	2.34
COQ9	1.56	1.52
COQ6	1.34	1.18
COQ7	1.34	1.64
COQ3	1.3	1.29
COQ10A	1.24	1.17

Structural

Gene	wt	h_{H49K}IF1
TIMM44	1.68	1.5
HSP70	1.66	1.4
VDAC1	1.61	1.26
VDAC2	1.45	1.17
HSP60	1.45	1.4

Soleus / Gastrocnemius ratio

Translocases

Gene	wt	h_{H49K}IF1
ATP2A2	2.76	2.64
ATP1B1	2.39	2.56
ABCB8	1.84	1.41
SLC25A20	1.8	1.63
SLC25A3	1.67	1.53
SLC27A1	1.67	1.6
LETM1	1.63	1.63
SLC25A11	1.57	1.61
SLC25A4	1.53	1.42
SLC25A5	1.53	1.25
ARALAR1	1.2	1.21

Lipid metabolism

Gene	wt	h_{H49K}IF1
FABP3	3.4	3.04
BDH1	2.61	3
ETFB	2.29	2.1
ACOT2	2.18	1.79
HADH	2.17	1.89
ECI2	2.17	1.61
HADHB	2.14	1.89
HSDL2	2.09	2.07
OXCT1	2.08	1.99
ACADM	2.08	1.9
ECI1	2.07	1.95
ACAA2	2.06	2.11
ETFA	2	1.85
HADHA	1.99	2.31
ACADL	1.99	1.56
ACAT1	1.95	1.94
ETFDH	1.92	1.77
ECHS1	1.78	1.99
ACSL1	1.76	1.56
CRAT	1.72	1.58
ACADS	1.67	1.46
HIBADH	1.63	1.75
ACAD12	1.63	1.6
ACADVL	1.59	1.69
CPT2	1.55	1.6
MMUT	1.53	1.59
IVD	1.52	1.49
CPT1B	1.47	1.54
DBI	1.47	1.31
APOO	1.45	1.38
ACOT9	1.41	1.16
HSD17B10	1.39	1.48
CYB5R3	1.38	1.02
DLD	1.37	1.19
PCCB	1.36	1.15
HMGCL	1.36	1.2
BCAT2	1.35	1.2
ACAD9	1.3	1.19
DBT	1.23	1.33
HIBCH	1.1	1.29
PCCA	1.07	1.11

Ratio: 0 | 0.5 | 1 | 2 | ≥4

Fig S4

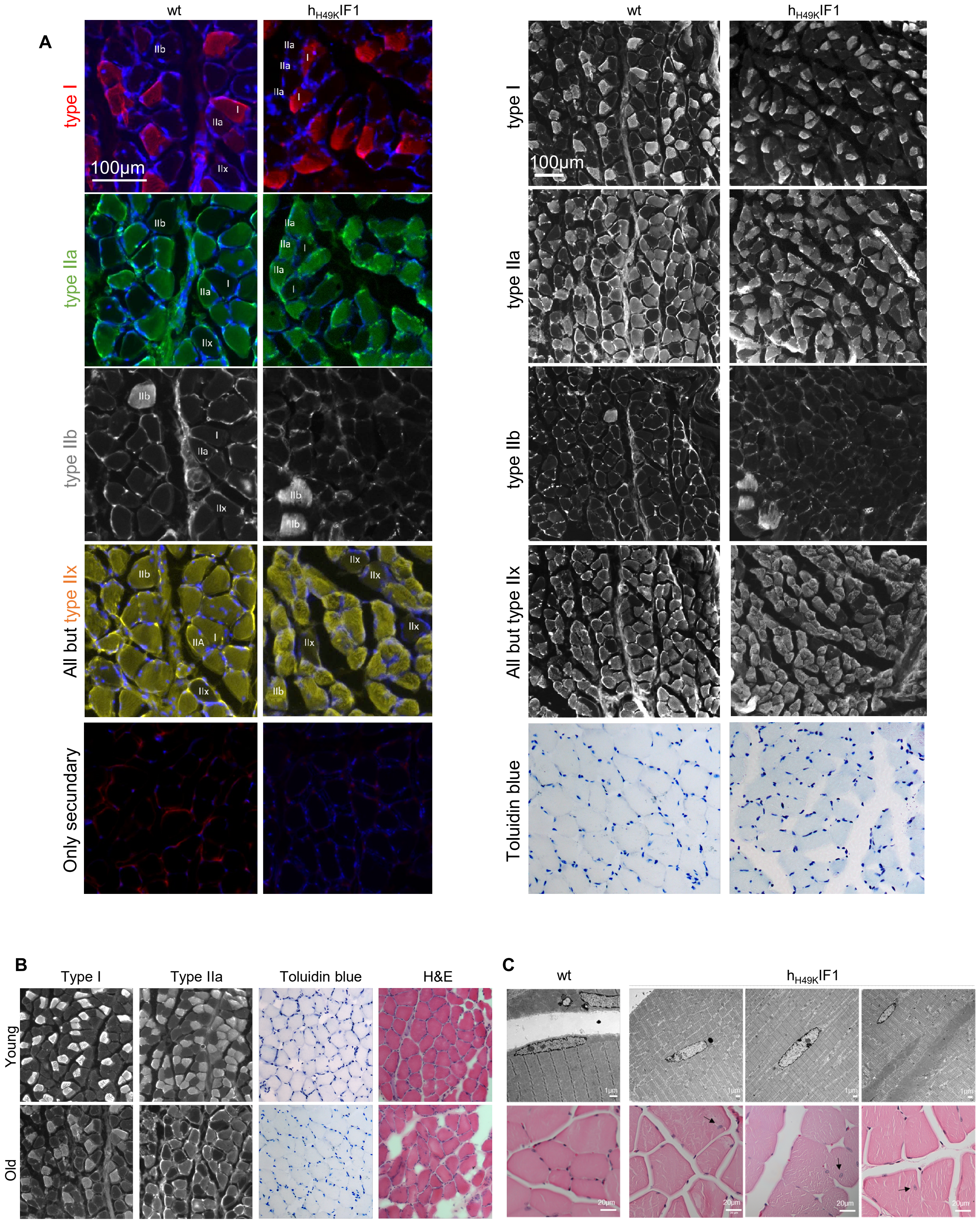
A
wt
hH49KIF1
type I
type IIa
type IIb
All but type IIx
Only secundary
Toluidin blue
100µm
IIb
IIa
IIx
I
IIA
B
Type I
Type IIa
Toluidin blue
H&E
Young
Old
C
wt
hH49KIF1
1µm
20µm

Fig S5

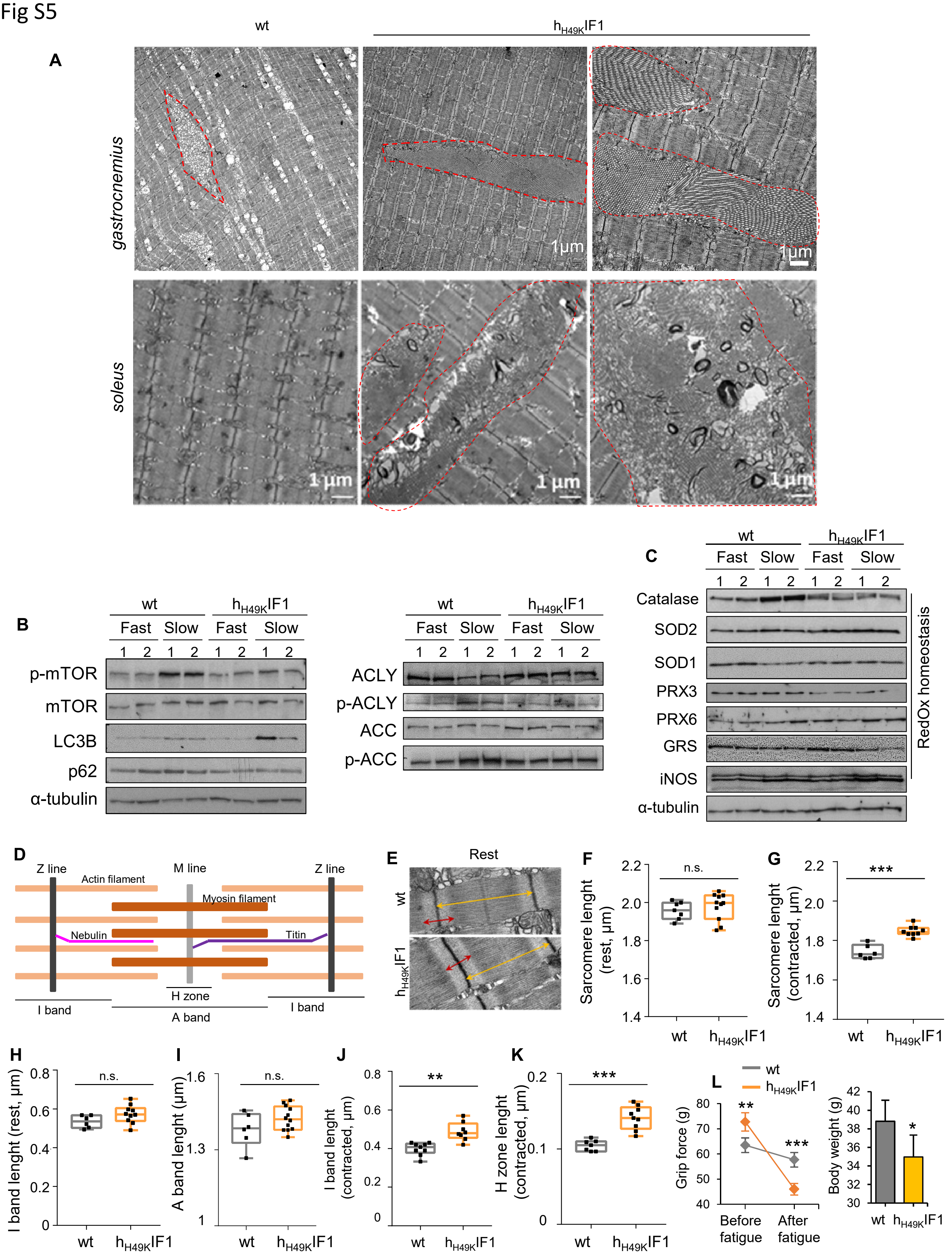

wt
h_{H49K}IF1
A
gastrocnemius
soleus
1µm
1 µm
B
C
wt
h_{H49K}IF1
Fast
Slow
1
2
p-mTOR
mTOR
LC3B
p62
α-tubulin
ACLY
p-ACLY
ACC
p-ACC
Catalase
SOD2
SOD1
PRX3
PRX6
GRS
iNOS
RedOx homeostasis
D
Z line
M line
Actin filament
Myosin filament
Nebulin
Titin
I band
H zone
A band
E
Rest
F
Sarcomere lenght (rest, µm)
n.s.
G
Sarcomere lenght (contracted, µm)
***
H
I band lenght (rest, µm)
I
A band lenght (µm)
J
I band lenght (contracted, µm)
**
K
H zone lenght (contracted, µm)
L
Grip force (g)
Before fatigue
After fatigue
Body weight (g)
*

Fig S6

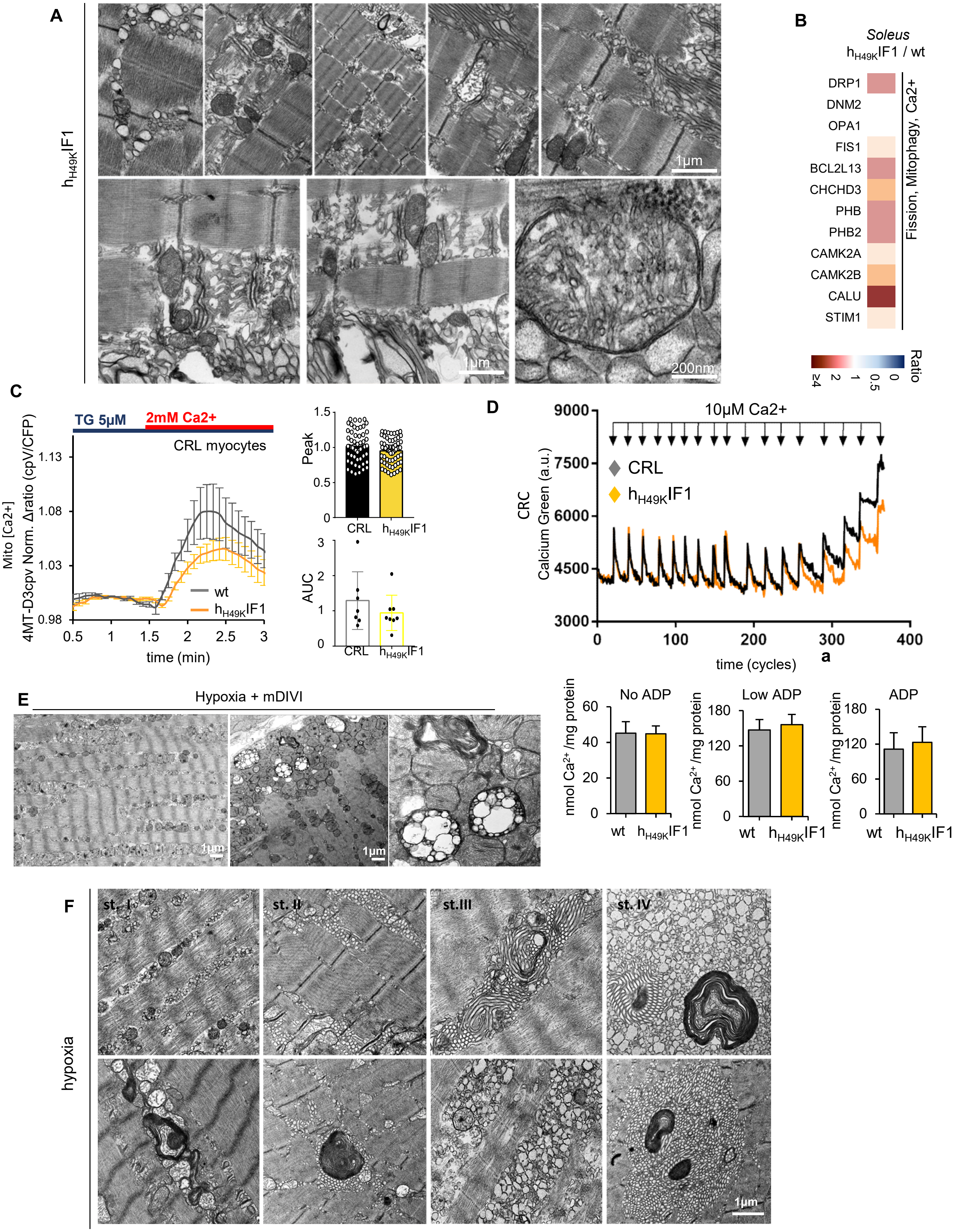

A
hH49KIF1
1µm
200nm
B
Soleus
hH49KIF1 / wt
DRP1
DNM2
OPA1
FIS1
BCL2L13
CHCHD3
PHB
PHB2
CAMK2A
CAMK2B
CALU
STIM1
Fission, Mitophagy, Ca2+
Ratio
≥4 2 1 0.5 0
C
TG 5µM
2mM Ca2+
CRL myocytes
Mito [Ca2+]
4MT-D3cpv Norm. Δratio (cpV/CFP)
wt
hH49KIF1
time (min)
Peak
AUC
CRL
hH49KIF1
D
10µM Ca2+
CRL
hH49KIF1
CRC
Calcium Green (a.u.)
time (cycles)
a
No ADP
Low ADP
ADP
nmol Ca2+ /mg protein
wt
hH49KIF1
E
Hypoxia + mDIVI
1µm
F
hypoxia
st. I
st. II
st.III
st. IV
1µm

Fig S7

A

edaravone

	gastrocnemius		*soleus*	
	wt	h_{H49K}IF1	wt	h_{H49K}IF1
ABCB8	1	1.03	1.29	1.4
ACAA2	1	1.12	2.85	2.7
ACAD12	1	1.03	1.29	1.4
ACAD9	1	1.16	1.26	1.34
ACADL	1	1.03	2.17	2.18
ACADM	1	1.09	2.65	2.79
ACADS	1	1.13	1.94	1.91
ACADVL	1	1.11	2.44	1.94
ACAT1	1	0.96	2.59	2.06
ACO2	1	1.18	1.84	1.7
ACOT1	1	0.83	1.79	5.74
ACOT13	1	1	1.75	2.12
ACOT2	1	1.08	2.5	2.66
ACSL1	1	1.08	2.17	2.26
AIFM1	1	1.19	1.83	2.01
ALDH2	1	0.99	1.67	1.72
ALDH4A1	1	1.06	1.31	1.28
ALDH6A1	1	0.96	1.34	1.29
APOO	1	1.08	1.23	1.47
APOOL	1	0.99	1.2	1.3
ATP5C1	1	1.12	1.7	1.77
ATP5F1A	1	1.18	1.7	1.72
ATP5F1B	1	1.15	1.75	2.08
ATP5F1C	1	1.12	1.7	1.77
ATP5F1D	1	1.1	1.62	1.86
ATP5F1E	1	1.05	1.28	1.36
ATP5ME	1	1.12	1.32	1.87
ATP5MF	1	1.06	1.39	2.18
ATP5MG	1	1.19	1.46	2.06
ATP5PB	1	1.08	1.61	1.79
ATP5PD	1	1.1	1.49	1.75
ATP5PO	1	1.04	1.5	1.65
ATP6V1A	1	1.07	1.19	1.17
ATP6V1B2	1	1.04	1.31	1.09
AUH	1	1.02	1.54	1.57
BCAT2	1	0.99	1.2	1.17
BCKDHA	1	1.02	1.44	1.57
BCL2L13	1	0.9	1.24	1.17
BDH1	1	1.06	6.01	6.68
BLVRB	1	1.04	0.9	1.22
CCDC51	1	1.2	1.3	1.78
CHCHD3	1	1.01	1.42	1.34
COQ3	1	1.04	1.33	1.52
COQ5	1	1.03	1.31	1.92
COQ6	1	1.1	1.45	1.5
COQ7	1	1.02	1.37	1.57
COQ7	1	1.02	1.37	1.57
COQ7	1	1.02	1.37	1.57
COQ8A	1	1.2	2.47	2.41
COQ9	1	1.02	1.39	1.41
COX4I1	1	0.81	1.09	1.43
COX5A	1	0.95	1.32	1.69
COX5B	1	0.9	1.09	1.35
COX5B-PS	1	0.9	1.09	1.35
COX6B1	1	0.97	1.07	1.16
COX6C	1	0.93	0.99	1.75
CPT1B	1	0.88	1.69	1.58
CPT2	1	1.05	1.95	1.84
CRAT	1	1.11	1.84	1.67
CYB5R1	1	1.1	1.06	1.3
CYB5R3	1	1.04	1.12	1.35
CYC1	1	1	1.53	1.52
CYCS	1	1.09	1.68	2.23
DBT	1	1.01	1.49	1.59
DGLUCY	1	1.12	1.11	1.86
DHODH	1	1.08	1.37	1.68
DLAT	1	1.26	1.42	1.46
DLST	1	1.11	1.52	1.51
ECH1	1	0.99	2.33	2.41
ECHS1	1	0.93	1.88	1.96
ECI1	1	1.11	2.32	2.65
ECSIT	1	1.21	1.42	1.36
ENDOG	1	1.12	1.32	1.61
ETFA	1	1.04	2.01	1.85
ETFB	1	1.03	2.25	2.57
ETFDH	1	1.15	2.6	2.31
FECH	1	1.04	1.2	1.17
FH	1	1.1	1.35	1.43
GATD3A	1	0.89	1.41	1.48
GCDH	1	1.74	1.57	4.08
GFM2	1	1.14	1.62	1.73
GLRX5	1	1.06	1.09	1.27
GLUD1	1	1.09	1.44	1.55
GPD2	1	1.18	0.73	0.73

edaravone

	gastrocnemius		*soleus*	
	wt	h_{H49K}IF1	wt	h_{H49K}IF1
GRPEL1	1	1.06	1.56	1.94
GSR	1	0.95	1.37	1.62
HADH	1	1.13	2.47	2.52
HADHA	1	1.11	3.56	2.76
HADHB	1	1.04	3.11	2.63
HAGH	1	1.05	1.02	1.09
HIBADH	1	1.03	1.37	1.49
HIBCH	1	1.06	1.36	1.46
HINT2	1	0.95	1.45	1.76
HSPD1	1	1.16	1.56	1.62
HSPE1	1	1.08	1.32	1.7
IARS2	1	1.2	1.36	1.54
IDH1	1	1	1.07	1.21
IDH2	1	0.93	4.55	4.22
IDH3A	1	1.03	1.33	0.98
IDH3B	1	1.1	1.24	1.21
IDH3G	1	1.18	1.5	1.38
LETM1	1	1.16	1.33	1.57
MAVS	1	0.93	1.43	1.64
MCCC1	1	1	1.36	1.3
MCCC2	1	1.09	1.46	1.46
MDH2	1	1.07	1.49	1.54
ME3	1	1.2	1.47	1.41
MLYCD	1	1.08	1.45	1.63
MMUT	1	1.02	1.56	1.49
MRPL1	1	1.1	1.02	1.12
MRPL39	1	1.11	1.48	1.87
MRPS27	1	1.1	1.12	1.32
MRPS34	1	1.18	1.3	1.64
MRPS35	1	1.19	1.14	1.23
MSRA	1	0.92	1.05	1.29
MTARC2	1	0.96	1.05	1.17
MTCO2	1	0.79	1.28	1.39
NDUFA10	1	0.96	1.18	1.01
NDUFA12	1	0.98	1.26	1.52
NDUFA12	1	0.98	1.26	1.52
NDUFA13	1	0.89	1.46	1.73
NDUFA2	1	0.79	1.26	1.51
NDUFA3	1	0.86	1.34	1.41
NDUFA4	1	0.94	1.29	1.72
NDUFA5	1	0.93	1.55	1.63
NDUFA6	1	0.93	1.7	1.87
NDUFA7	1	0.9	1.26	1.29
NDUFA8	1	0.96	1.49	1.44
NDUFA9	1	0.97	1.59	1.56
NDUFA9	1	0.97	1.59	1.56
NDUFAB1	1	1.06	1.39	1.93
NDUFAF1	1	1.08	1.14	1.48
NDUFB10	1	0.96	1.63	1.88
NDUFB11	1	0.75	1.64	1.57
NDUFB3	1	0.95	1.5	1.81
NDUFB4	1	0.96	1.4	1.61
NDUFB5	1	0.91	1.84	1.99
NDUFB6	1	1.03	1.55	1.96
NDUFB7	1	0.99	1.48	1.74
NDUFB8	1	0.99	1.53	1.82
NDUFB9	1	0.96	1.42	1.53
NDUFC2	1	0.9	1.54	1.65
NDUFS1	1	1	1.73	1.96
NDUFS2	1	1.06	1.47	1.42
NDUFS3	1	0.92	1.54	1.48
NDUFS4	1	0.92	1.4	1.56
NDUFS4	1	0.92	1.4	1.56
NDUFS5	1	1	1.4	1.56
NDUFS6	1	0.91	1.41	1.55
NDUFS7	1	0.9	1.47	1.37
NDUFS8	1	0.99	1.46	1.46
NDUFV1	1	1	1.72	1.7
NDUFV2	1	0.93	1.43	1.49
NFS1	1	1.03	1.14	1.23
NFU1	1	1.16	1.44	1.78
NNT	1	1.07	1.95	2.92
NUDT9	1	1.08	1.42	1.44
OAT	1	1.06	1.03	1.08
OGDH	1	1.3	1.89	1.88
OPA1	1	1.06	1.34	1.33
OXCT1	1	1	2.32	2.52
PC	1	1	0.93	1.23
PCCA	1	1.33	1.58	1.59
PCCB	1	1.24	1.43	1.57
PDHA1	1	1.15	1.29	1.32
PDHB	1	1.15	1.44	1.69
PDHX	1	1.17	1.37	1.42
PDK2	1	1.07	1.47	1.59

edaravone

	gastrocnemius		*soleus*	
	wt	h_{H49K}IF1	wt	h_{H49K}IF1
PDK4	1	1.45	1.75	2.82
PDP1	1	1.01	1.26	1.17
PDPR	1	1.11	1.48	1.43
PITRM1	1	1.1	1.32	1.44
PMPCA	1	0.96	1.03	0.97
PMPCB	1	1.05	1.27	1.5
PPA2	1	1.04	1.38	1.62
PRDX3	1	1.13	1.36	1.56
PRDX5	1	0.99	1.57	2.01
REXO2	1	0.93	1.02	1.17
RTN4IP1	1	1.09	1.28	1.57
SDHA	1	1	1.74	1.62
SDHB	1	0.97	1.56	1.67
SHMT2	1	1.11	1.4	1.58
SLC25A11	1	1.06	1.7	1.86
SLC25A12	1	1.21	1.42	1.45
SLC25A20	1	0.84	2.02	2.02
SLC25A3	1	0.68	1.44	1.16
SOD2	1	1.02	1.52	1.64
SQOR	1	1.08	0.72	0.79
SSBP1	1	0.97	1.13	1.15
STOML2	1	1	1.23	1.31
SUCLA2	1	1.13	1.46	1.39
SUCLG1	1	1.02	1.26	1.21
SUCLG2	1	1.12	1.28	1.42
SUOX	1	1.1	1.19	4.12
TFAM	1	0.95	0.99	1.01
TIMM44	1	1.03	1.25	1.38
TMEM11	1	1.12	1.09	0.87
TOMM70	1	1.05	0.94	0.99
TRAP1	1	1.19	1.43	1.52
TSFM	1	0.83	1.13	1.3
TUFM	1	1.05	1.26	1.39
TXN2	1	1.07	1.71	2.28
UQCC2	1	1.11	1.08	1.34
UQCR10	1	1.02	1.31	1.81
UQCRB	1	1.02	1.71	2.02
UQCRC1	1	1.02	1.63	1.62
UQCRC2	1	1.02	1.73	1.53
UQCRFS1	1	0.94	1.73	1.66
UQCRQ	1	1.08	1.44	2.14
VDAC1	1	0.87	1.21	1.11
VDAC2	1	0.93	1.09	1.01
VDAC3	1	0.69	1.07	1.02

B

edaravone

	gastrocnemius		*soleus*	
	wt	h_{H49K}IF1	wt	h_{H49K}IF1
HK2	1	1.05	1.44	1.46
GPI	1	1.06	0.84	0.71
PFKM	1	0.76	0.79	0.71
ALDOART1	1	1.03	0.78	0.83
ALDOART2	1	1.04	0.72	0.79
TPI1	1	0.95	0.73	0.72
GPD1	1	0.97	0.6	0.63
PGK1	1	1.03	0.77	0.73
PGM1	1	1.08	0.84	0.79
PGM5	1	1.13	0.97	1.01
PGAM2	1	0.93	0.65	0.63
ENO1	1	0.85	0.91	0.88
ENO2	1	1.07	0.63	0.6
ENO3	1	1	0.78	1.01
LDHA	1	0.96	0.71	0.75
PYGM	1	1.15	0.89	0.72

\+ -

Relative expression

Fig S8

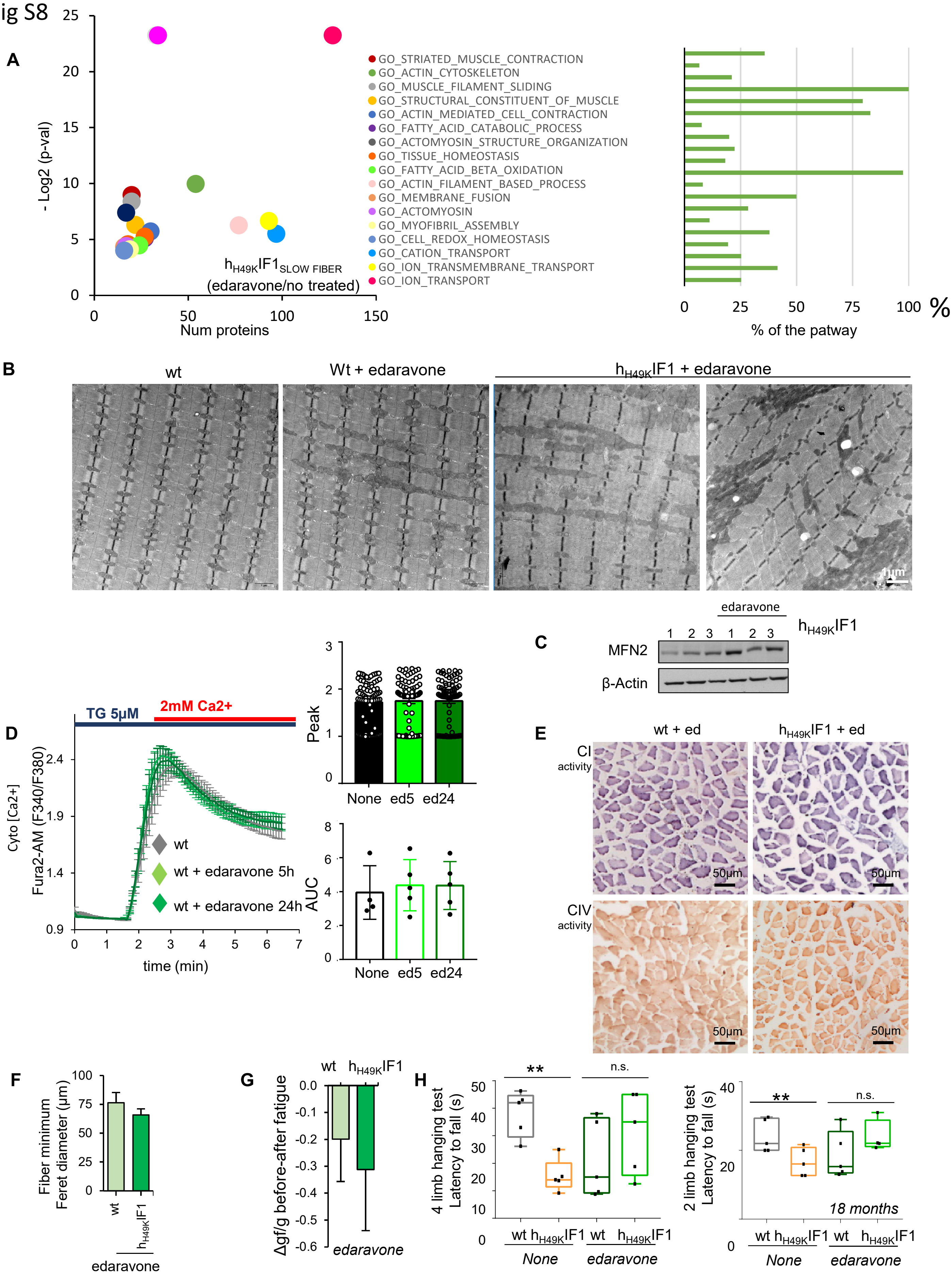
A
- Log2 (p-val)
Num proteins
hH49KIF1SLOW FIBER
(edaravone/no treated)
GO_STRIATED_MUSCLE_CONTRACTION
GO_ACTIN_CYTOSKELETON
GO_MUSCLE_FILAMENT_SLIDING
GO_STRUCTURAL_CONSTITUENT_OF_MUSCLE
GO_ACTIN_MEDIATED_CELL_CONTRACTION
GO_FATTY_ACID_CATABOLIC_PROCESS
GO_ACTOMYOSIN_STRUCTURE_ORGANIZATION
GO_TISSUE_HOMEOSTASIS
GO_FATTY_ACID_BETA_OXIDATION
GO_ACTIN_FILAMENT_BASED_PROCESS
GO_MEMBRANE_FUSION
GO_ACTOMYOSIN
GO_MYOFIBRIL_ASSEMBLY
GO_CELL_REDOX_HOMEOSTASIS
GO_CATION_TRANSPORT
GO_ION_TRANSMEMBRANE_TRANSPORT
GO_ION_TRANSPORT
% of the patway
B
wt
Wt + edaravone
hH49KIF1 + edaravone
1µm
C
edaravone
hH49KIF1
MFN2
β-Actin
D
TG 5µM
2mM Ca2+
Cyto [Ca2+]
Fura2-AM (F340/F380)
time (min)
wt
wt + edaravone 5h
wt + edaravone 24h
Peak
None ed5 ed24
AUC
E
wt + ed
hH49KIF1 + ed
CI activity
CIV activity
50µm
F
Fiber minimum Feret diameter (µm)
wt
hH49KIF1
edaravone
G
Δgf/g before-after fatigue
edaravone
H
4 limb hanging test
Latency to fall (s)
2 limb hanging test
Latency to fall (s)
**
n.s.
18 months
None
edaravone

Fig S9

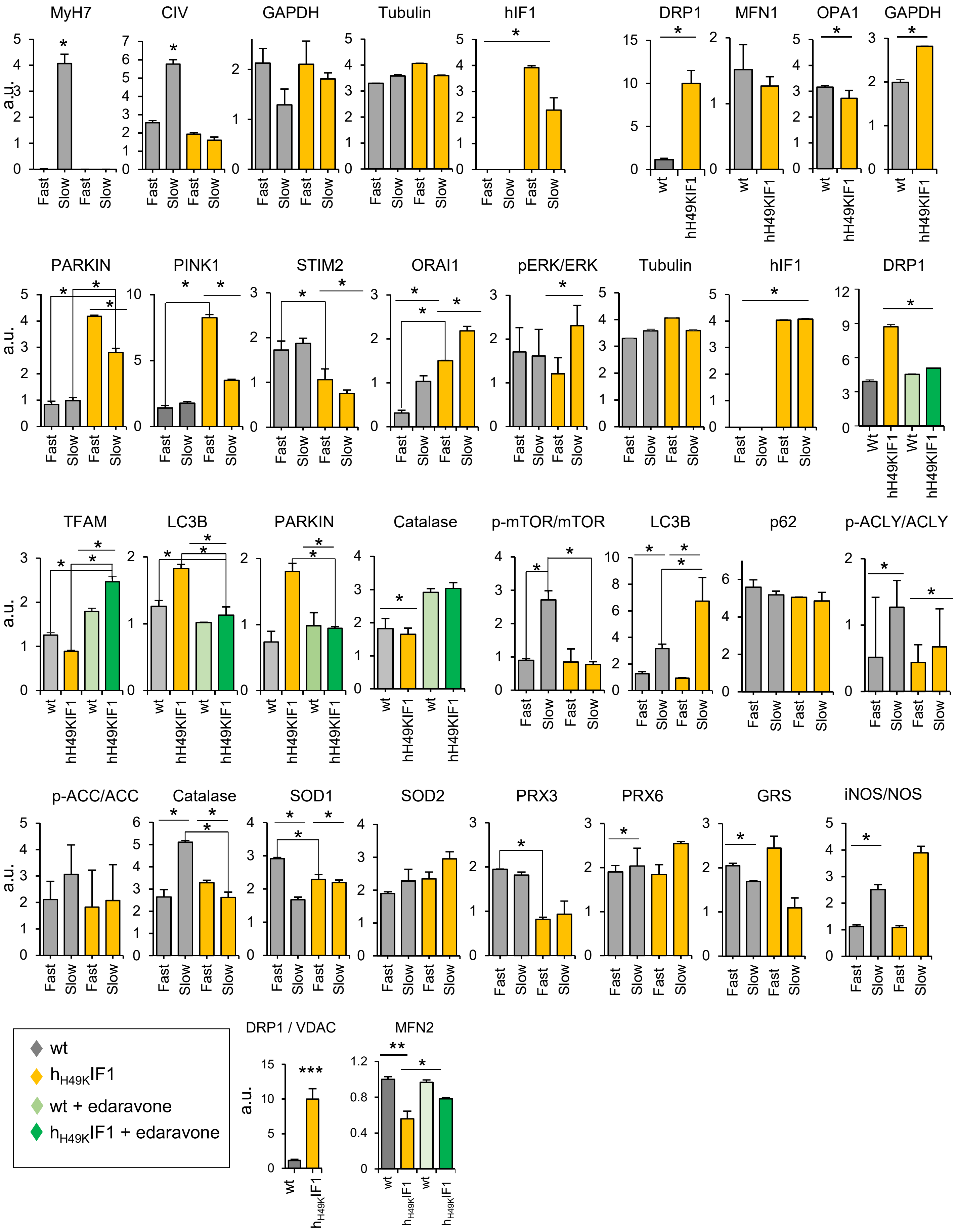

MyH7
CIV
GAPDH
Tubulin
hIF1
DRP1
MFN1
OPA1
GAPDH
PARKIN
PINK1
STIM2
ORAI1
pERK/ERK
Tubulin
hIF1
DRP1
TFAM
LC3B
PARKIN
Catalase
p-mTOR/mTOR
LC3B
p62
p-ACLY/ACLY
p-ACC/ACC
Catalase
SOD1
SOD2
PRX3
PRX6
GRS
iNOS/NOS
DRP1 / VDAC
MFN2
a.u.
Fast
Slow
wt
hH49KIF1
wt
h_{H49K}IF1
wt + edaravone
h_{H49K}IF1 + edaravone

Supplementary Figures

Fig. S1. Reverse Phase Protein Array of Skm TAM samples. ***Related to Fig. 1***

Reverse Phase Protein Array (RPPA) of Skm biopsy extracts from healthy (CRL, dark blue; n=11), TAM (yellow; n=6), MELAS (red; n=4) and PEO (light blue, n=4) patients. The arrays were developed with the indicated primary antibodies (See also Fig 1A).

Mitochondrial proteins from (i) OXPHOS (SDHA, SDHB, CoreII, COXIV and β-F1 ATPase); (ii) mitochondrial dynamics (OPA1, MFN1, MFN2); (iii) FAO (CPT1, HADHA, ETFA, ETFB) and (iv) structure (HSP60) are presented. Moreover, proteins from glucose metabolism (GAPDH, MDH2) and redox system (catalase, SOD2, PRX3, PRX6 and GR) are also shown. Linear plot of the mouse muscle C2C12 cell line was used as loading control. Bars indicate the mean ± SEM of 3 replicates of the n above indicated.

In red, the proteins significantly down-regulated in TAM.

*$p < 0.05$ when compared CRL by ANOVA and Student's t-test.

Fig. S2. Skm protein perturbations in restrained OXPHOS mice. ***Related to Fig. 1,2***

A) Representative images of Skm muscles from 18-year-old wt and h_{H49K}IF1 mice. In the lower panel, *gastrocnemious* images from the two genotypes.

B) Quantitative proteomic analysis (TMT) of soleus (slow) from wt (n=4) and h_{H49K}IF1 (n=4) mice. Volcano plot compares soleus proteins from h_{H49K}IF1 and wt animals. On the right the GSEA bioinformatics analysis of TMT results. Dot charts of normalized enrichment score (NES) of 20 significantly altered pathways in *soleus* muscles from h_{H49K}IF1 mice compared to wt. Dot color and size represent p-values and the number of altered proteins in the pathway, respectively.

C) GSEA- analysis of 20 perturbed pathways in h_{H49K}IF1 vs wt mice. Chart on the left correlates the number of affected proteins with p-value. Histograms in the right show the percentage of alteration for each pathway.

D) Chart show most upregulated (orange) and downregulated (green) proteins in *soleus* muscle from h_{H49K}IF1 mice compared to wt.

E) qPCR relative expression of proteins related to Skm fiber type. Values are expressed as h_{H49K}IF1/wt ratio, wt, n = 4; h_{H49K}IF1, n = 4. Relative expression of MYH7, MYH1, Troponin T1 (TNNT1), Tropomyosin 3 (TPM3), Calsequestrin 1 and 2 (CASQ1 and 2), Actinin alpha 3 (ACTN3) and Myosin-binding protein H (MYBPH) is shown.

F) Contiguous transversal slices from h_{H49K}IF1 Skm stained for mitochondrial complex I activity (left) and immuno-labeled for fiber-type specific MYHs (right). Images are representative of 6 slices/condition, wt, n = 3; h_{H49K}IF1, n = 4.

Fig. S3. Glycolytic and mitochondrial proteins in h_{H49K}IF1 mice. ***Related to Fig. 1***

Quantitative proteomic analysis (TMT) values for proteins from glycolysis and mitochondria in *soleus* and *gastrocnemius* muscles from wt and h_{H49K}IF1 mice. Higher intensities of brown or green colours represent higher or lower h_{H49K}IF1/wt expression ratios, respectively. n = 4 animals/genotype.

Fig. S4. Alteration in fiber composition and myonuclear organization upon ATP synthase inhibition. ***Related to Fig. 1, 2***

A. Transversal slices of *soleus* from wt and h_{H49K}IF1 18 months-old mice immuno-labeled for antibodies against specific fiber-type MYHs. Type I fibers (MYH7, antibody clone: BA-F8); type IIa fibers (MYH2, antibody clone: SC-71); type IIb fibers (MYH4, antibody clone: DF-F3); type IIx fibers (antibody clone: BF-35, all but type IIx fibers). DAPI stained nuclei. Secundary andibody (no primary antibody) and toluidine blue staining are also shown.

wt, n = 3; h_{H49K}IF1, n = 3;

B. Transversal slices of *soleus* from young (6 months-old) and old (18 months old) wt mice immuno-labeled for antibodies against type I fibers; type IIa fibers; toluidine blue and haematoxylin/eosin staining.

C. Representative TEM images (upper panels) and hematoxylin/eosin staining (lower panels) in longitudinal (upper panels) or transversal (lower panels) *soleus* slices from wt and h_{H49K}IF1 18 months-old mice. Images are representative of n=6 mice/genotype; 10 images/mice

Fig. S5. Sarcomere disorganization upon ATP synthase inhibition. ***Related to Fig. 2***

A) Representative TEM images of Skm tissue in transversal slices of *gastrocnemius* (upper panels) and *soleus* (lower panels) from wt and hH49KIF1 18 months-old mice. Red lines delimitates the TA area. Images are representative of n=4 mice/genotype.

B) Representative WB expression of markers of autophagy and *de novo* lipogenesis enzymes in *soleus* (slow) and *gastrocnemius* (fast) muscles from wt and hH49KIF1. LC3B, microtubule-associated proteins 1A/1B light chain 3B; p62, polyubiquitin chain binding protein; p-mTOR, phospho- mechanistic target of rapamycin; mTOR, mechanistic target of rapamycin. ACLY (ATP citrate synthase) and pACLY (phospho ATP citrate synthase), ACC (acetyl-CoA carboxylase) and pACC (phospho acetyl-CoA carboxylase. Tubulin is shown as loading control. Two samples per condition, each sample contains protein extracts from 3 mice. Quantification in Fig. S8.

C) Representative WB expression of redox system proteins in *soleus* (slow) and *gastrocnemius* (fast) muscles from wt and hH49KIF1. Catalase; SOD2, superoxide dismutase 2; SOD1, superoxide dismutase 1; PRX·, peroxiredoxin 3; PRX6, peroxiredoxin 6; GRS, glutathione reductase; iNOS, nitric oxide synthase Tubulin is shown as loading control. Two samples per condition, each sample contains protein extracts from 3 mice. Quantification in Fig S8.

D) Schematic representation of the sarcomere structure (I band, A band and H zone) in which the main proteins are represented (myosin, brown; actin, light orange; nebulin, pink; and titin, purple).

E) Representative TEM images of transversal slices of rested *soleus* from wt and h_{H49K}IF1 18 months-old mice. Images are representative of n=4 mice/genotype; 10 images/mice

F-K) Sarcomere (F,G), A band (I), I bands (H,J) and H zone (K) length in *soleus* from wt and h_{H49K}IF1 mice. Measures are taken in 10 images/mouse. Plots are the mean±SEM of n=4 animals/genotype.

L) Motor behaviour assays. Grip-force test before and after 1 hour rotarod fatigue in 18 months old mice (wt, n = 7; h_{H49K}IF1, n = 6). Histograms show the weight of the animals. Bars are the mean ± SEM of the reported n.

*, **, *** $p < 0.05, 0.01, 0.001$ when compared to wt by ANOVA and Student's t-test, respectively.

Fig. S6. Mitochondrial/SR contact sites, calcium fluxes and TA in wt and h_{H49K}IF1 mice. ***Related to Fig 4.***

A) Representative TEM images of transversal slices of *soleus* from wt and h_{H49K}IF1 18 months-old mice. Images are representative of n=4 mice/genotype; 10 images/mice. Note the mitochondria/SR contact sites and the mitochondrial connection with TA clusters.

B) TMT h_{H49K}IF1/wt ratio of proteins related to mitochondrial fission, mitophagy and calcium homeostasis in *soleus* muscles from wt and mice (DRP1; DNM2, dynamin-related-2; OPA1; FIS1, mitochondrial fission 1 protein; BCL2L13, BCL2 Like 13; CHCHD3, MICOS19; PHB, prohibitin; PHB2, prohibitin 2; CAMK2, calcium/calmodulin dependent protein kinase II; CALU, calumenin; STIM1).

C) Mitochondrial calcium uptake in C_2C_{12} myocytes expressing or not h_{H49K}IF1. Curves are the mean ± SEM of 3 experiments, n=10 cells/genotype. Histograms on the right represent Peak and Under Curve Area (AUC) in control (CRL) and h_{H49K}IF1 expressing myocytes.

D) Calcium Retention Capacity (CRC) in isolated Skm mitochondria from wt and h_{H49K}IF1mice (15 sec/cycle). Histograms on the right represent the amount of calcium per mg of mitochondrial proteins in the presence or absence of ADP. Bars are the mean ± SEM of 3 animals, n=10 traces/genotype.

E) Representative TEM images of transversal slices of *soleus* from wt 18 months-old mouse muscles after 6h hypoxia in the presence of the DRP1 inhibitor mDIVI. Images are representative of n=4 mice/genotype; 10 images/mice. Note larger mitochondria but also strong mitochondrial swelling when fission is inhibited under hypoxia.

F) Representative TEM images of *ex-vivo* muscles after hypoxia. State I, II, III and IV show mitochondria adjacent to SR tubules. TA appeared to generate in close contact with

dysfunctional mitochondria until organelles are surrounded and degraded (st IV) and only SR tubules remain. n=4 muscle/genotype; 10 images/mouse.

Fig. S7. Glycolytic and mitochondrial proteins in edaravone-treated wt and h_{H49}K mice. ***Related to Fig. 5***

A,B) Quantitative proteomic analysis (TMT) values for proteins from mitochondria (A) and glycolysis (B) in *soleus* and *gastrocnemius* muscles from wt and h_{H49K}IF1 mice after 2 months of edaravone treatment. Higher intensities of red or blue colours represent higher or lower expression levels, respectively. n = 4 animals/genotype.

Fig. S8. Effects of edaravone treatment on Skm. ***Related to Fig. 6***

A) Bioinformatic analysis of TAM proteomics in Fig. 4. GSEA-analysis of 20 perturbed pathways in edaravone-treated vs no treated h_{H49K}IF1 mice. Chart on the left correlates the number of affected proteins with p-value. Histograms in the right show the percentage of alteration for each pathway.

B) TEM images of mitochondrial population in transversal slices of *soleus* from wt and h_{H49K}IF1 18 months-old mice treated or not with edaravone. Images are representative of n=4 mice/genotype; 10 images/mice

C) Representative WB expression of mitofusin 2 in *soleus* from h_{H49K}IF1 animals treated or not with edaravone. 3 samples per condition, each sample contains protein extracts from 3 mice.

D) Cytosolic calcium uptake measured in C_2C_{12} myocytes treated or not with edaravone for 5 or 24h. Bars are the mean ± SEM of 3 experiments, n=10 cells/genotype. Histograms below represent Peak and Under Curve Area (AUC) in control (CRL) and h_{H49K}IF1 expressing myocytes.

E) Transversal slices of *soleus* from wt and h_{H49K}IF1 18 months-old mice treated with edaravone stained for mitochondrial complex I (CI) or IV (CIV) activities. Images are representative of 6 slices/condition, n=4 mice/genotype.

F) Fiber minimum Feret diameter in wt and h_{H49K}IF1 18 months-old mice treated with edaravone. Bars are the mean ± SEM of n=6 measurement/genotype from images in E

G-H) Motor behaviour assays. wt, n=4; h_{H49K}IF, n=4; wt + edaravone= 5; h_{H49K}IF1 + edaravone, n=5. (G) Grip-force test before and after 1 hour rotarod fatigue in 18 months old mice Histograms show the Δg/g (force/weight) after-before exercise. (H) Latency to fall in 4

limb and 2 limb hanging tests in 6 months- and 18 months-old animals. Bars are the mean ± SEM of the reported n.

In D, H, box plots represent 25th to 75th percentiles with the median value in the middle line, and with all data represented from minimal to maximal values.

*, **, *** $p < 0.05$; 0.01 and 0.001 when compared to wt by ANOVA and Student's t-test, respectively.

Fig. S9. WB quantifications. ***Related to Fig. 1-6***

Quantification of WB protein expression in *gastrocnemius* (fast) and *soleus* (slow) muscles in wt and h_{H49K}IF1 18-month-old mice treated or not with edaravone. Bars are the mean ± SEM of n = 6 animals/genotype/age.

* $p < 0.05$ when compared to wt by ANOVA and Student's t-test.

Supplementary Tables

REAGENTS	SOURCE	RRID	REFERENCE
**Antibodies**			
Anti-ACC	Cell Signaling Technology Inc	AB_2219400	3662
Anti-ACLY	Abcam	AB_722533	ab40793
Anti-Catalase	Sigma-Aldrich	AB_258720	C0979
Anti-COXIV	Abcam	AB_301443	ab14744
Anti-DRP1	BD Transduction Laboratories	AB_398423	611112
Anti-FASN	BD Transduction Laboratories	AB_398275	610962
Anti-GAPDH	Homemade	-	-
Anti-GSR	Abcam	AB_2115640	ab16801
Anti-mouse IF1	Homemade	-	-
Anti-human IF1(H49K)	Homemade	-	-
Anti iNOS/NOS	BD Transduction Laboratories	AB_397718	610328
Anti-LC3B	AB_2137703		4108
Anti-MFN1	Abcam	AB_2142624	ab57602
Anti-MFN2	Abcam	AB_2142629	ab56889
Anti-Mouse IgG (H+L), HRP Conjugate	Promega	AB_430834	W4021
Anti-mTOR	Cell Signaling Technology Inc	AB_330978	2972
Anti-MyHC	Abcam	AB_297734	ab11083
Anti-OPA1	BD Transduction Laboratories	AB_399888	612606
Anti ORAI1	alomone labs	AB_10918021	ACC-062
Anti-p62	Enzo Life Science	AB_2052149	BML-PW9860-0025
Anti-pACC	Cell Signaling Technology Inc	AB_330337	3661
Anti-pACLY	Cell Signaling Technology Inc	AB_2257987	4331
Anti-Parkin	Abcam	AB_1566559	ab77924
Anti-PINK1	Abcam	AB_447627	ab23707
Anti-Phospho-mTOR (Ser2448)	Cell Signaling Technology Inc	AB_330970	2971
Anti-PRX3	Abcam	-	ab222807

Anti-PRX6	Abcam	AB_944762	ab59543
Anti-Rabbit IgG (H+L), HRP Conjugate	Promega	AB_430833	W4011
Anti-SOD1	Santa Cruz Biothecnology Inc	AB_2193779	sc11407
Anti-SOD2	Santa Cruz Biothecnology Inc	AB_661470	sc30080
Anti-STIM2	Alomone labs	AB_2040218	ACC-064
MYHs:			
MYH7, type I fibers	Developmental Studies Hybridoma Bank.	-	Clone BA-F8
MYH2, type IIa fibers	Developmental Studies Hybridoma Bank.	-	Clone SC-71
MYH4, type IIb fibers	Developmental Studies Hybridoma Bank.	-	Clone BF-F3
All but type IIx fibers	Developmental Studies Hybridoma Bank.	-	Clone BF-35
Anti-tubulin	Sigma-Aldrich	AB_477579	T5168
Donkey anti-goat	Thermo Fisher	-	A-21447
Donkey anti-mouse	Sigma-Aldrich	-	SAB4600176-250UL
Goat anti-rabbit	Sigma-Aldrich	-	SAB4600185-250UL
**Chemicals, Peptides and Recombinant Proteins**			
2-isopropanol	Merk KGaA	-	K48640034
6-aminohexanoic acid	Sigma-Aldrich	-	7260
ABI Prism 7900HT sequence detection system	Thermo Fisher Scientific	-	4317596
Acetic acid	Sigma-Aldrich	-	71251
ADP	Sigma-Aldrich	-	A2754
Antimycin A	Sigma-Aldrich	-	A8474
ATP	Sigma-Aldrich	-	A2383
Bio-Rad Protein Assay	Bio-Rad	-	5000006
Bis-Tris	Sigma-Aldrich	-	T0377
Bovine Serum Albumin (BSA)	Nzytech	-	MB04602
Bromophenol Blue	Sigma-Aldrich	-	B0126
Carnitine	Sigma-Aldrich	-	C0283

cOmpleteTM Mini EDTA-free Protease Inhibitor Cocktail	Sigma-Aldrich	-	11836170001
Digitonin	Sigma-Aldrich	-	D5628
DNP	Sigma-Aldrich	-	D198501
Doxycyling	Sigma-Aldrich	-	D9891
DTT	Sigma-Aldrich	-	D9760
Edaravone	Selleckchem	-	S1326
EDTA	Sigma-Aldrich	-	ED2P
EGTA	Sigma-Aldrich	-	E3889
EtOH	Merck KGaA	-	64-17-5
Etomoxir	Sigma-Aldrich	-	E1905
EZ View Red Protein G Affinity Gel	Sigma-Aldrich	-	E3403
Fast Green FCF	Sigma-Aldrich	-	F7252
Fast SYBERMasterMix	Thermo Fisher	-	4385616
FCCP	Sigma-Aldrich	-	C2920
FDA-Approved library	Selleckchem	-	L1300
Fetal Bovine Serum (FBS)	Sigma-Aldrich	-	F7524
Fura-2AM	Invitrogen	-	F1221
Glycine	VWR	-	101196X
Glucose	Merck KGaA	-	K37462374
Glutamate	Sigma-Aldrich	-	49621
Glycerol	Sigma-Aldrich	-	G5516
IAM	Sigma-Aldrich	-	I6125
Insulin	Sigma-Aldrich	-	I6634
KCl	Merk	-	104936
Lipofectamine 3000 Transfection Reagent	Thermo Fisher Scientific	-	L3000015
Malate	Sigma-Aldrich	-	2300
Malonate	Sigma-Aldrich	-	M1296
mDIVI	Selleckchem	-	S7162
Metanol (MetOH)	Sigma-Aldrich	-	34860
$MgCl_2$	Sigma-Aldrich	-	M8266
$MgSO_4$	Sigma-Aldrich	-	M7506
MitoSOX Red Mitochondrial superoxide indicator	Invitrogen	-	M36008
Mowiol	Merck KGaA	-	475904-100GM

Non-fatty dried milk	Central lechera Asturiana	-	
Novex ECL HRP Chemiluminiscent reagent	Invitrogen	-	WP20005
Oligomycin	Sigma-Aldrich	-	O4876
Paraformaldehyde	Santa Cruz Biotechnology	-	30525-89-4
Phosphatase inhibitor cocktail 2	Sigma-Aldrich	-	P5726
Pluronic acid	Invitrogen	-	24040032
Ponceau Red	Sigma-Aldrich	-	P7170
Probenecid	Sigma-Aldrich	-	P8761
Rotenone	Sigma-Aldrich	-	R8875
Sodium Deoxycolate	Sigma-Aldrich	-	D6750
Sodium Palmitate	Sigma-Aldrich	-	P9767
Sodium Succinate	Sigma-Aldrich	-	S7501
Sucrose	Sigma-Aldrich	-	84100
Super G blocking buffer	Grace Bio-labs	-	105101
TPER Buffer	Thermo Fisher Scientific	-	78501
Tricine	Sigma-Aldrich	-	B9754
Tris pH 8.3	Sigma-Aldrich	-	T1503
TritonX-100	Merck KGaA	-	9030-19-5
Trizol	Invitrogen	-	15596026
Tween 20	EMD Millipore Corp.	-	817072
Xylene	VWR	-	1330-20-7
**Critical Commercial Assays**			
Glycerol Quantification Kit	Sigma-Aldrich	-	T2449
Glycerol Quantification Kit	Sigma-Aldrich	-	F6428
High-Capacity cDNA Reverse Transcription Kit	Thermo Fisher	-	4368814
KAPA Mousse Genotyping Kit	Kapa Biosystems	-	KK7302
**Recombinant DNA**			
H49KhIF1 plasmid		-	
pcDNA-lynD3cpv	Addgene		37472
pcDNA-4mtD3cpv	Addgene		36324
**Software and Algorithms**			
Cytoscape v3.6.1	Cytoscape Consortium	SCR_015784	

GraphPad Prism7	GraphPad	SCR_002798	
GSEA v3.0	Broad Institute Inc.	SCR_003199	
Image J analyssis software	NIH	SCR_003070	
PEAKS Studio X search enginw	Bioinformatics Solutions Inc.	-	
Seahorse_Wave_Desktop_v2.4	Agilent technologies	SCR_014526	
SPSS 17.0	SPSS Inc.	SCR_002865	
**Other**			
BEAD MILL 24 homogenizer	Fisherbrand	-	15515799
Bead mill 24 Beads	Fisherbrand		15515809
iTWO-300 RPPA microarray	Axi-Vend	-	-
Nitrocellulose membrane, Amersham Protran 0.2mm NC	GE Healthcare	-	
PVDF membrane, Immobilon-P, 0.45uM	Merck KGaA	-	IPVH00010
XF24 Flux Pack, Seahorse Bioscience	Agilent Technologies	-	100867-100
XFe96 Flux Pack, Seahorse Bioscience	Agilent Technologies	-	102416-100
Reinforced bulk tubes	Fisherbrand		15535809

PRIMERS:

FGF21	Fw:	CTGGGGGTCTACCAAGCATA
	Rv:	CACCCAGGATTTGAATGACC
MYG	Fw:	CCTGGGTACCATCCTGAAGA
	Rv:	AAAGTCCCCGGAATGTCTCT
MyoD	Fw:	CTCCAACTGCTCTGATGGCA
	Rw:	GAGATGCGCTCCACTATGCT
PGC1α	Fw:	TTGCTAGCGGTTCTCACAGA
	Rw:	TAAGACCGCTGCATTCATTG
PPARα	Fw	TCTGGAAGCTTTGGTTTTGC
	Rv	TTCGACACTCGATGTTCAGG
PPARδ	Fw:	CAGAATTCCTCCCCTTCCTC
	Rv:	TTGCGGTTCTTCTTCTGGAT
PPARγ	Fw:	AAGAGCTGACCCAATGGTTG

	Rv:	GCATCCTTCACAAGCATGAA
PRDM16	Fw:	GAAGTCACAGGAGGACACGG
	Rw:	TCGGCTCCAAAGCTAACAGG
Atp5b	Fw:	CCTGCTGATGACCTGACTGA
	Rw:	GCTGGATAGATGCCCAACTC
ND4	Fw:	AACGGATCCACAGCCGTA
	Rw:	AGTCCTCGGGCCATGATT
SDHA	Fw:	TACTACAGCCCCAAGTCT
	Rw:	CCGTGAAGACCTCAGCAACT
B2M	Fw:	CCGGATTGGCTGTGAGTT
	Rw:	GACAAGCACCAGAAAGACCAG
TNNT3	Fw:	CCCTCATTGACAGCCACTTT
	Rv:	CCTCTCTTCTGGCCTTCTCC
TNNT1	Fw:	TGCACTAAAAGACCGCATTG
	Rv:	TCTTCTTGGCGTCATCCTCT
TPM3	Fw:	ATCCAGCTGGTTGAAGAGGA
	Rv:	TCTGCAATGTGCTTTGCTTC
TPM1	Fw:	GCTGGTGTCACTGCAAAAGA
	Rv:	TCCAACTCCTCCTCAACCAG
MyH1	Fw:	AAGACCGAAGGCGGAACTAC
	Rv:	TGACAGTGACGCAGAACAGG
MYH7	Fw:	CTCAAGCTGCTCAGCAATCTATTT
	Rv:	GGAGCGCAAGTTTGTCATAAGT
MYBPH	Fw:	AGAATGGGTGCCTGTGAATC
	Rv:	GGGCTTCAGTGATCTCTTGG
aACTN-3	Fw:	AAATTGTTGACGGGAACCTG
	Rv:	TACGTTGACATTGCGGTACG

Table S1: Comprehensive list of reagents, primers and antibodies utilized in this study.
