# Supplementary material for: Chronic inhibition of the mitochondrial ATP synthase in skeletal muscle triggers sarcoplasmic reticulum distress and tubular aggregates
Source: Cell Death Dis. 2022 Jun 22;13(6):561. doi: 10.1038/s41419-022-05016-z (PMC9217934; doi:10.1038/s41419-022-05016-z)
Supplement: Supplementary file 3 — Uncropped WB [file 41419_2022_5016_MOESM3_ESM.pdf]

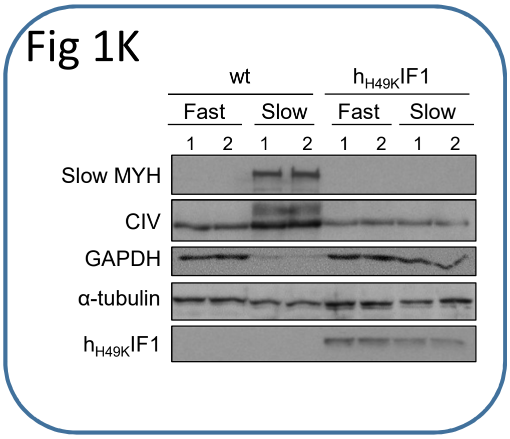

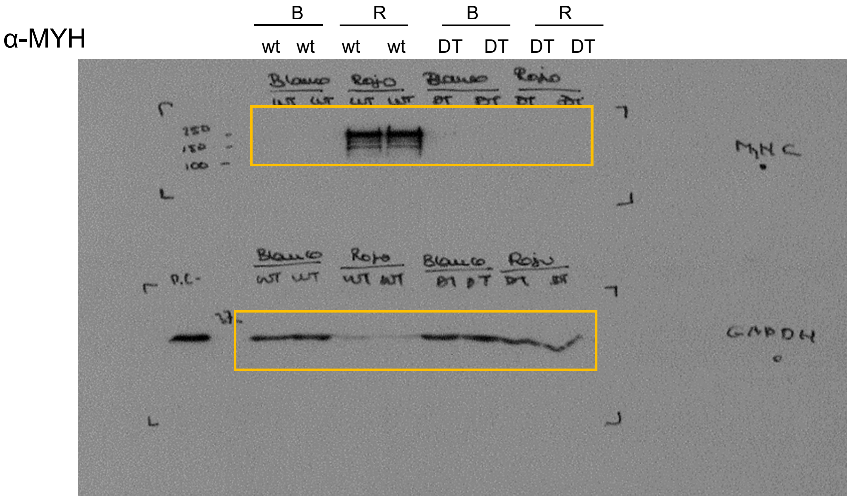

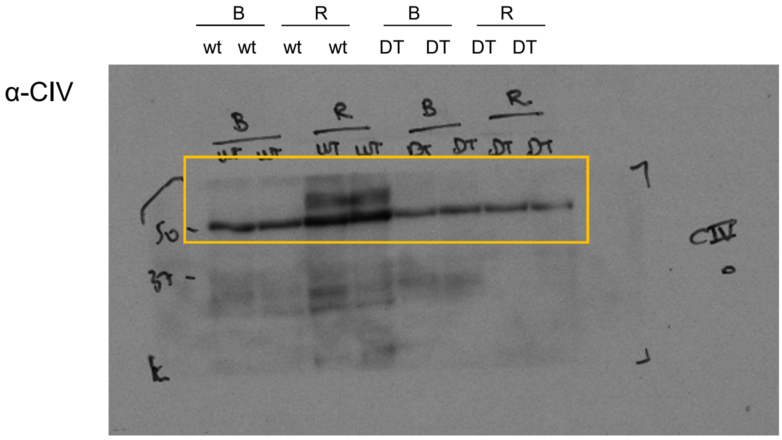

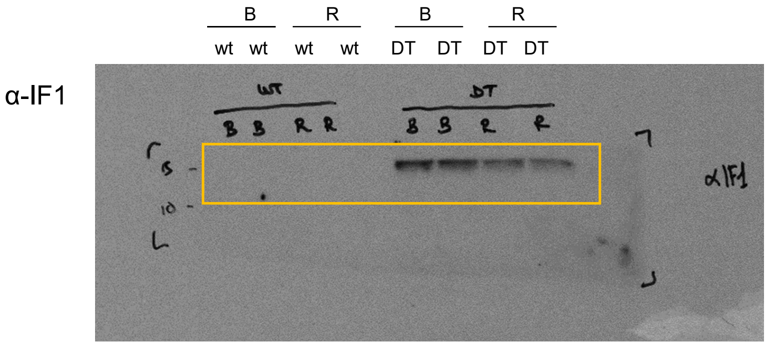

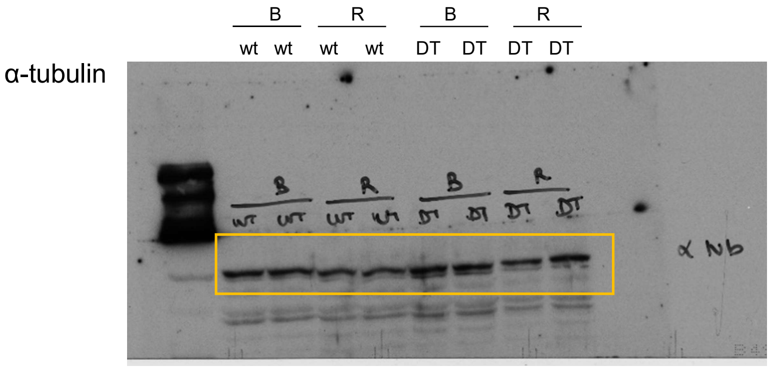

wt= wild type
DT= double transgenic: h_{H49K}IF1

B= fast glycolytic= *gastrocnemius*
R= red= slow oxidative= *soleus*

Fig 3F

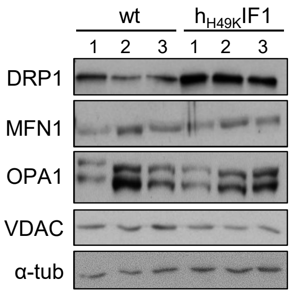

wt wt wt DT DT DT

α-DRP1

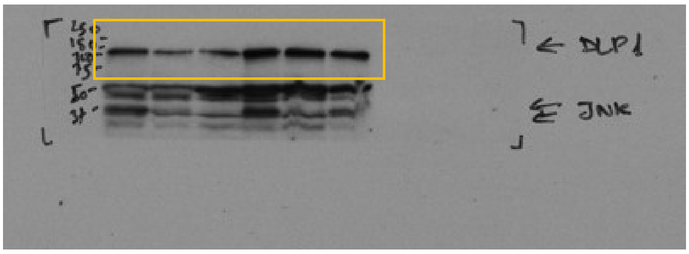

α-MFN1

wt wt wt DT DT DT

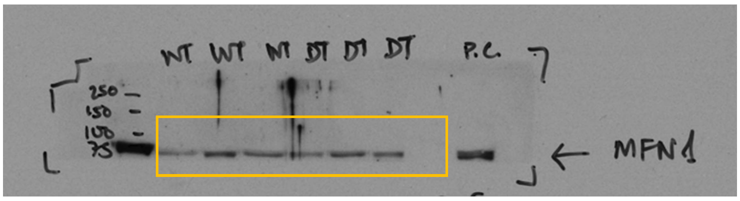

wt wt wt DT DT DT

α-OPA1

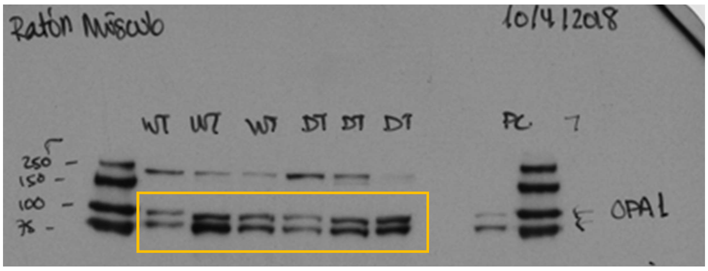

wt wt wt DT DT DT

α-tubulin

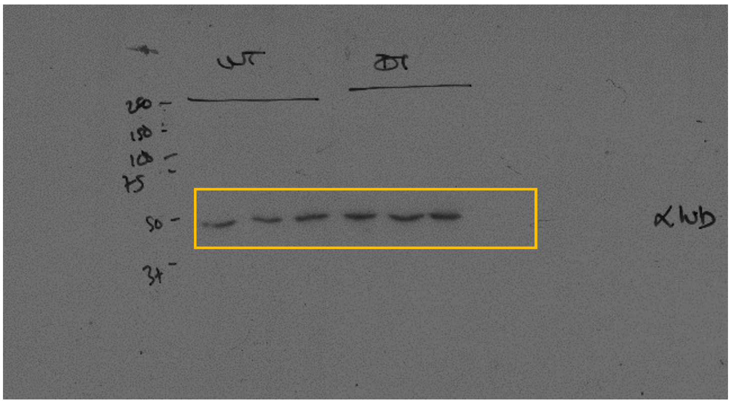

wt wt wt DT DT DT

α-VDAC

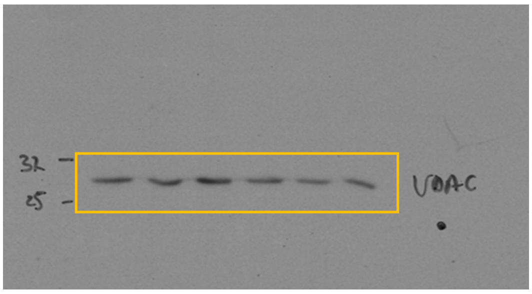

wt= wild type
DT= double transgenic: h_{H49K}IF1

Fig 3H

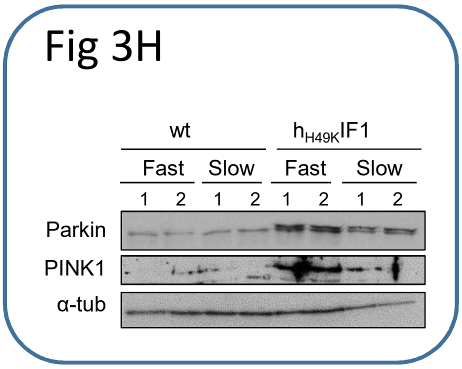

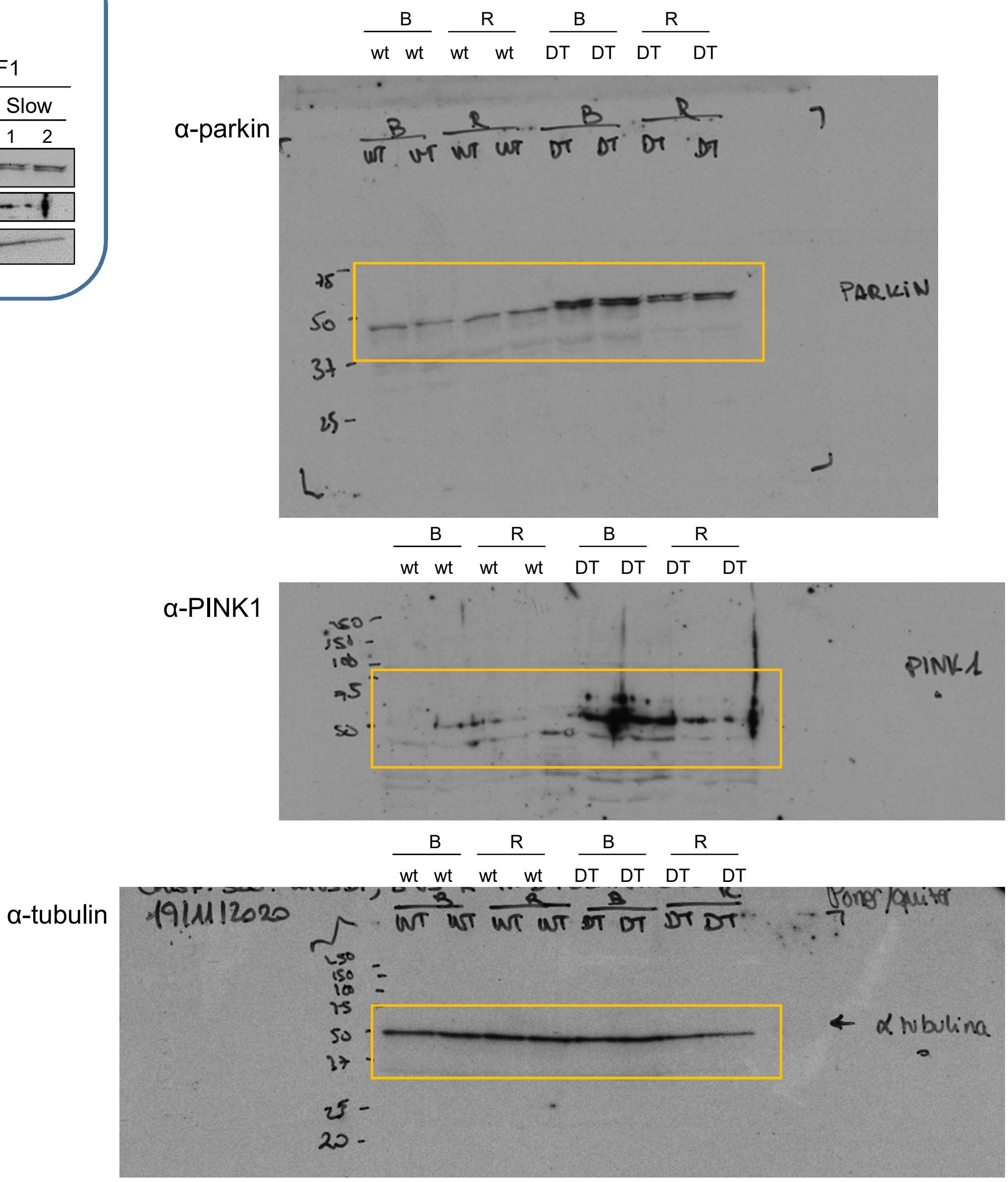

wt= wild type
DT= double transgenic: h_{H49K}IF1

B= fast glycolytic= *gastrocnemius*
R= red= slow oxidative= *soleus*

Fig 4A

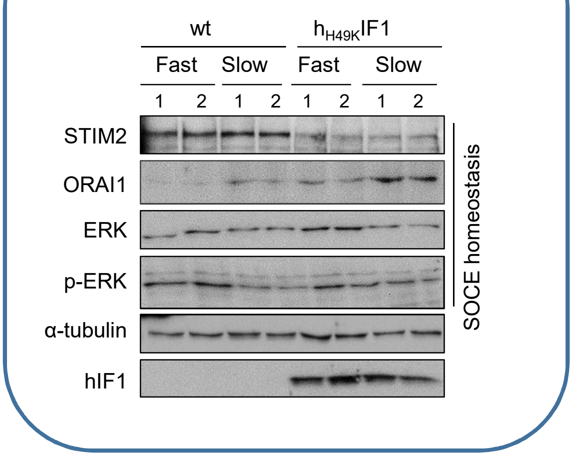

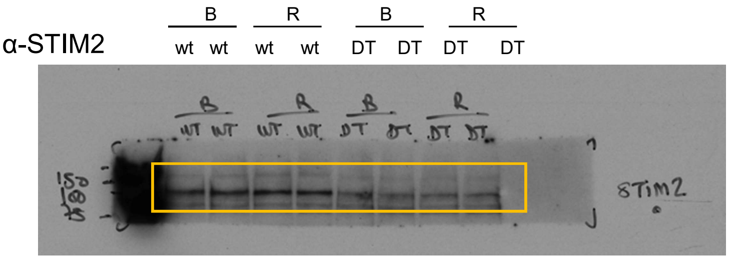

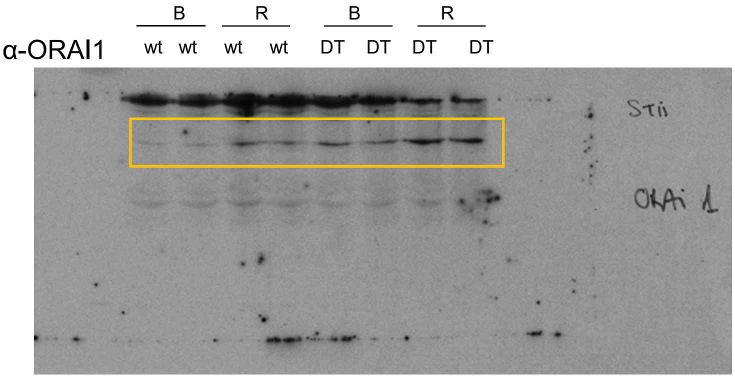

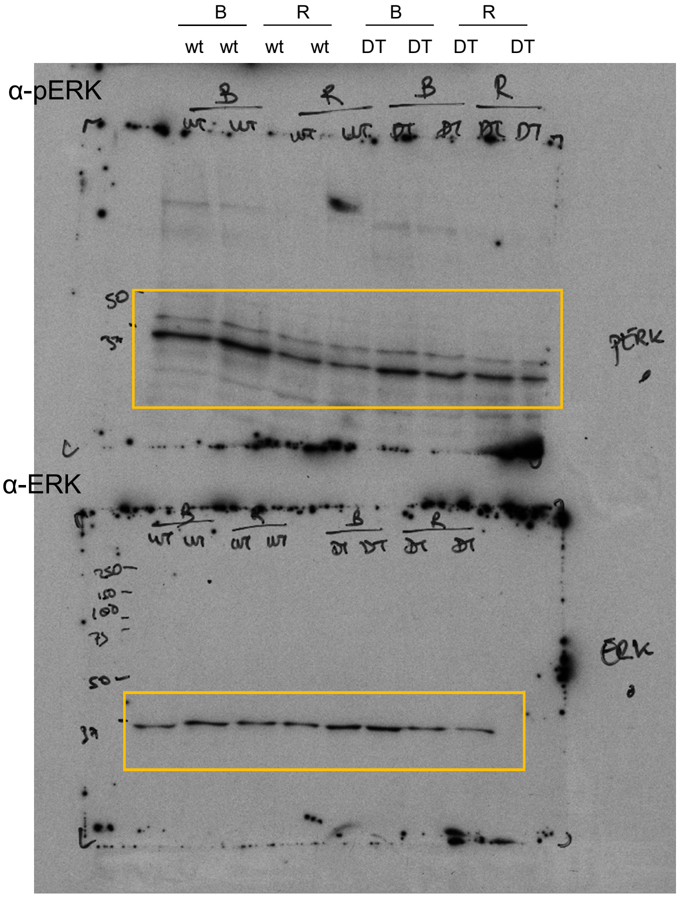

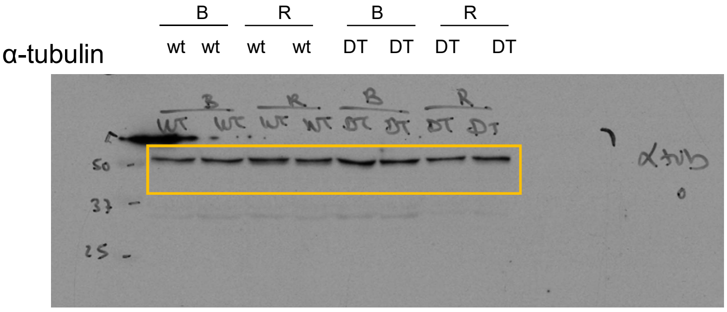

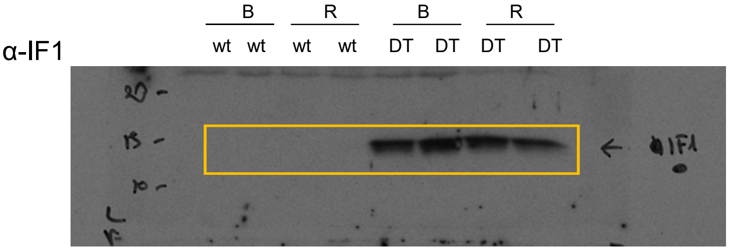

wt= wild type
DT= double transgenic: $h_{H49K}IF1$

B= fast glycolytic= *gastrocnemius*
R= red= slow oxidative= *soleus*

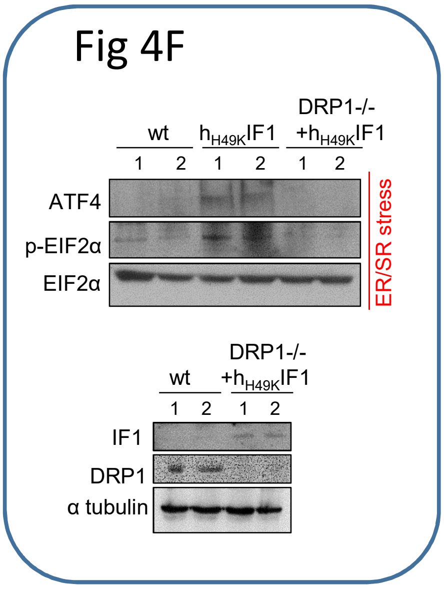

α-ATF4

wt wt DT DT DT-D DT-D

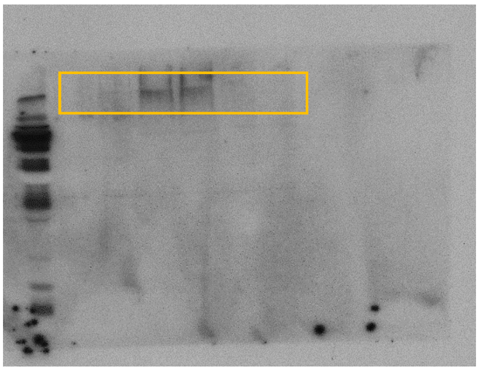

α-p-EIF2α

wt wt DT DT DT-D DT-D

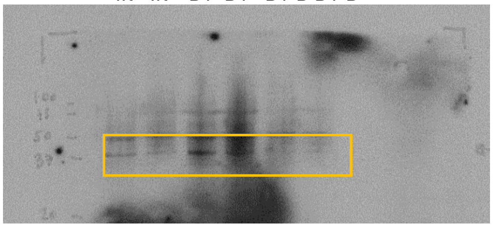

α-DRP1

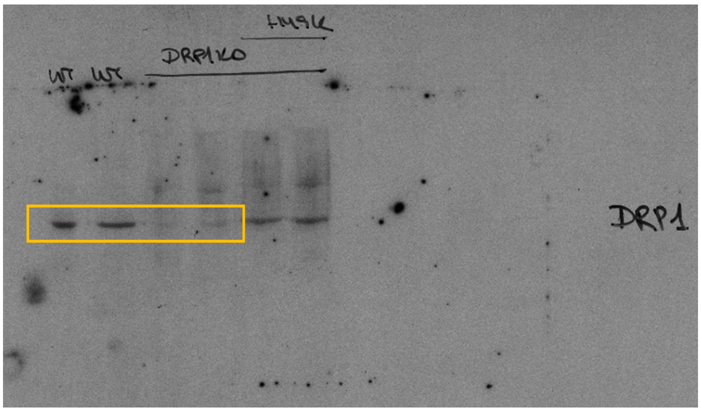

α-IF1

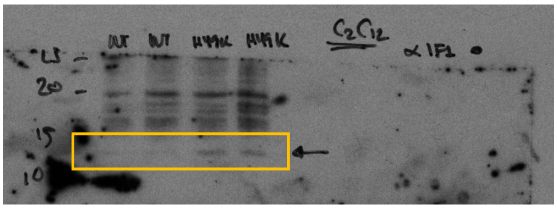

α-tubulin

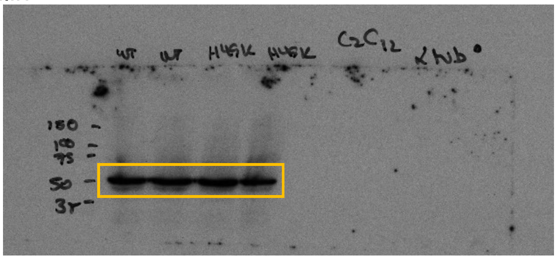

wt= wild type
DT= double transgenic: h_{H49K}IF1
DT-D= h_{H49K}IF1 DRP1-/-

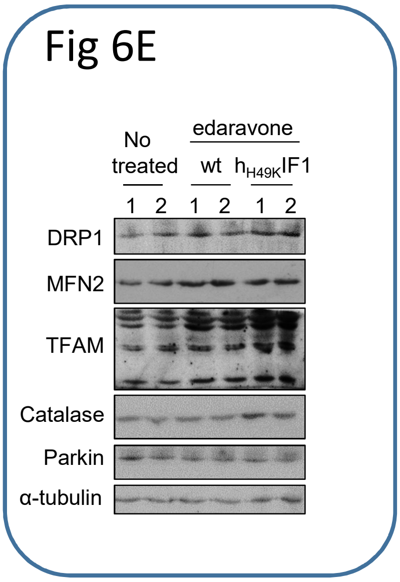

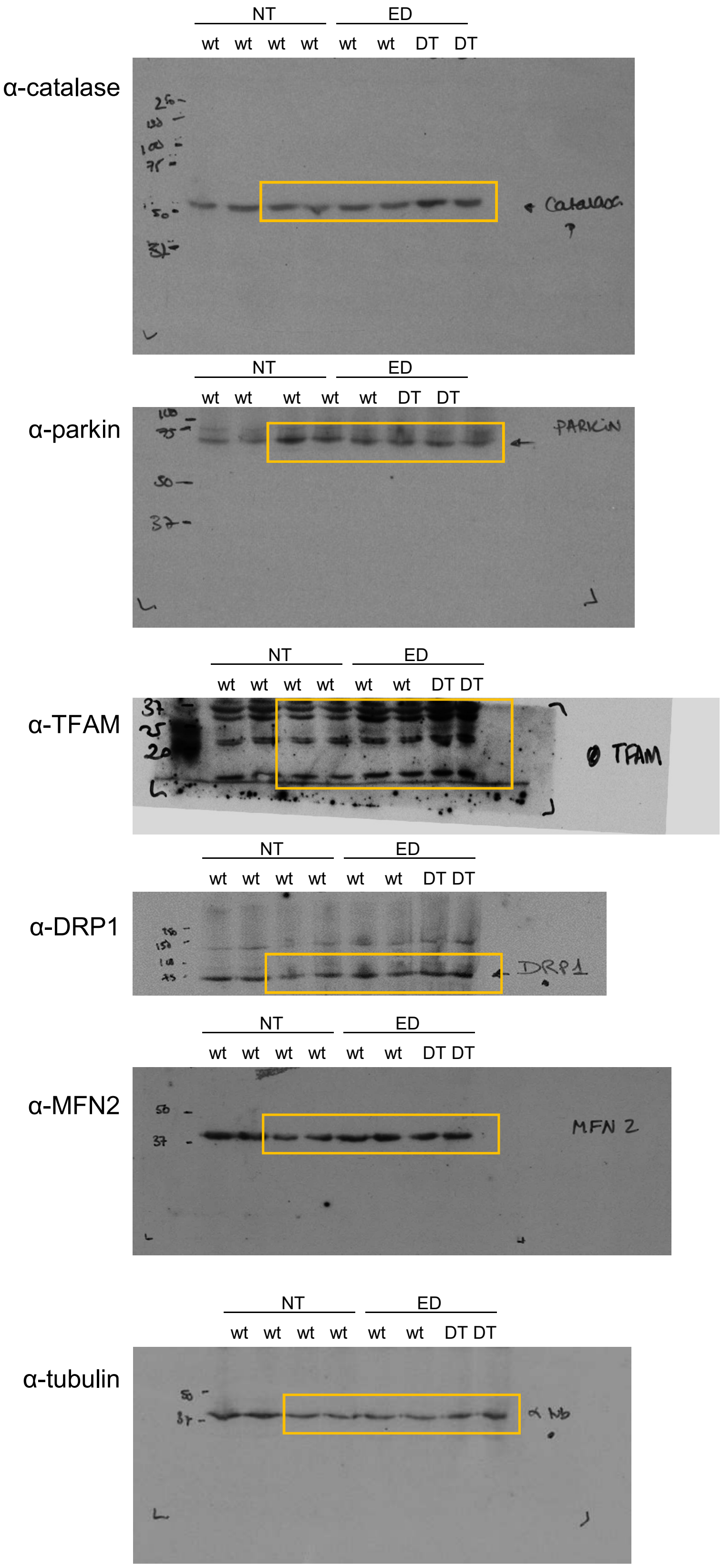

NT=no treated
ED= edaravone
wt= wild type
DT= double transgenic: h$_{H49K}$IF1
