# Supplementary material for: Chronic inhibition of the mitochondrial ATP synthase in skeletal muscle triggers sarcoplasmic reticulum distress and tubular aggregates
Source: Cell Death Dis. 2022 Jun 22;13(6):561. doi: 10.1038/s41419-022-05016-z (PMC9217934; doi:10.1038/s41419-022-05016-z)
Supplement: Supplementary file 4 — Agreement with final author list [file 41419_2022_5016_MOESM4_ESM.pdf]

Asunto: Re: CDDIS-21-4142RRR Initial Quality Check-URGENTE
De: Cristina Sánchez González <crissg31093@gmail.com>
Fecha: 08/06/2022 15:56
Para: Laura Formentini <lformentini@cbm.csic.es>
CC: Cristina Sánchez <cristina.sanchez@cbm.csic.es>, JUAN CRUZ HERRERO MARTIN <jherrero@cbm.csic.es>, cnunez@cbm.csic.es, Laura Contreras Balsa <lcontreras@cbm.csic.es>, "JM. Cuezva" <jmcuezva@cbm.csic.es>

Good afternoon,

Since their collaboration was fundamental, I totally agree with the final author list.

Best regards,

Cristina

Cristina Sánchez González

PhD in Molecular Biosciences

MBA in Pharma and Biotech Industries

El mié, 8 jun 2022 a las 15:52, Laura Formentini (<lformentini@cbm.csic.es>) escribió:

> As all of you already know, the work of Beñat Salegi Ansa, Brina Stancic and Marta P. Pereira was fundamental for answering Reviewer comments during revision.
> Cell Death and Disease request agreement from all authors for their addition into the final author list.
>
> Please, confirm that you agree to this final author list:
>
> "Sánchez-González, Cristina; Herrero Martín, Juan Cruz; Salegi Ansa, Beñat; Núñez de Arenas, Cristina; Stančič, Brina; Pereira P., Marta; Contreras, Laura; Cuezva, José M.; Formentini, Laura."
>
> Just replay to this email as your earliest convenience,
> Best regards,
> Laura
>
> -------- Mensaje reenviado --------
> **Asunto:** CDDIS-21-4142RRR Initial Quality Check
> **Fecha:** Wed, 8 Jun 2022 09:03:16 -0400
> **De:** cddisease@springernature.com
> **Responder a:** cddisease@springernature.com
> **Para:** lformentini@cbm.csic.es
>
> Dear Professor Formentini,
>
> In checking in your manuscript submitted to Cell Death & Disease it has come to our attention that the following must be addressed before we can proceed.
>
> 1. It has come to our attention that your most recent author list differs from the one in your original submission. We find that the following authors have been added since your initial submission: {Beñat Salegi Ansa; Brina Stančič; Marta P. Pereira}
>
> Please request agreement from all authors including additions and deletions, these can be collected in the following way:
>
> Email your co-authors with the change, and ask them to reply to your email confirming that they agree to these changes. Once you have collected these replies, please combine all of the co-authors' email responses in one document and upload this file to your submission.
>
> Your paper is now available for you to edit, you may access via the following link:
>
> https://mts-cddis.nature.com/cgi-bin/main.plex?el=A4CA2VyA1D5jSG5F3A9ftdsEGa8i7g6u5KZNVt5GaAZ
>
> (Press/Click on the above link to be automatically sent to the web page.)
>
> Please make the correction(s) as specified above and resubmit your paper following the same steps as before.
>
> If you have any questions please feel free to contact us.
>
> Sincerely,
>
> Editorial Office
> Cell Death & Disease
> cddisease@springernature.com
>
> This email has been sent through the NPG Manuscript Tracking System NY-610A-NPG&MTS
>
> *Confidentiality Statement:*
>
> *This e-mail is confidential and subject to copyright. Any unauthorised use or disclosure of its contents is prohibited. If you have received this email in error please notify our Manuscript Tracking System Helpdesk team at http://platformsupport.nature.com .*
>
> *Details of the confidentiality and pre-publicity policy may be found here http://www.nature.com/authors/policies/confidentiality.html*
>
> Privacy Policy | Update Profile

Asunto: Re: CDDIS-21-4142RRR Initial Quality Check-URGENTE
De: JUAN CRUZ HERRERO MARTIN <jherrero@cbm.csic.es>
Fecha: 08/06/2022 15:57
Para: Laura Formentini <lformentini@cbm.csic.es>
CC: Cristina Sánchez <cristina.sanchez@cbm.csic.es>, Cristina Sánchez González <crissg31093@gmail.com>, cnunez@cbm.csic.es, Laura Contreras Balsa <lcontreras@cbm.csic.es>, "JM. Cuezva" <jmcuezva@cbm.csic.es>

I completely agree with the final author list.

Best,
Herrero Martín, Juan Cruz

Laura Formentini <lformentini@cbm.csic.es> escribió:

> As all of you already know, the work of Beñat Salegi Ansa, Brina Stancic and Marta P. Pereira was fundamental for answering Reviewer comments during revision.
> Cell Death and Disease request agreement from all authors for their addition into the final author list.
>
> Please, confirm that you agree to this final author list:
> "Sánchez-González, Cristina; Herrero Martín, Juan Cruz; Salegi Ansa, Beñat; Núñez de Arenas, Cristina; Stančič, Brina; Pereira P., Marta; Contreras, Laura; Cuezva, José M.; Formentini, Laura.^"
>
> Just replay to this email as your earliest convenience,
> Best regards,
> Laura
>
> -------- Mensaje reenviado --------
> Asunto: CDDIS-21-4142RRR Initial Quality Check
> Fecha: Wed, 8 Jun 2022 09:03:16 -0400
> De: cddisease@springernature.com
> Responder a: cddisease@springernature.com
> Para: lformentini@cbm.csic.es
>
> Dear Professor Formentini,
>
> In checking in your manuscript submitted to Cell Death & Disease it has come to our attention that the following must be addressed before we can proceed.
>
> 1. It has come to our attention that your most recent author list differs from the one in your original submission. We find that the following authors have been added since your initial submission: {Beñat Salegi Ansa; Brina Stančič; Marta P. Pereira}
>
> Please request agreement from all authors including additions and deletions, these can be collected in the following way:
>
> Email your co-authors with the change, and ask them to reply to your email confirming that they agree to these changes. Once you have collected these replies, please combine all of the co-authors' email responses in one document and upload this file to your submission.
>
> Your paper is now available for you to edit, you may access via the following link:
>
> https://mts-cddis.nature.com/cgi-bin/main.plex?el=A4CA2VyA1D5jSG5F3A9ftdsEGa8i7g6u5KZNVt5GaAZ
>
> (Press/Click on the above link to be automatically sent to the web page.)

Please make the correction(s) as specified above and resubmit your paper following the same steps as before.

If you have any questions please feel free to contact us.

Sincerely,

Editorial Office
Cell Death & Disease
cddisease@springernature.com

This email has been sent through the NPG Manuscript Tracking System NY-610A-NPG&MTS

/Confidentiality Statement:

This e-mail is confidential and subject to copyright. Any unauthorised use or disclosure of its contents is prohibited. If you have received this email in error please notify our Manuscript Tracking System Helpdesk team at http://platformsupport.nature.com . /

/Details of the confidentiality and pre-publicity policy may be found here http://www.nature.com/authors/policies/confidentiality.html /

Privacy Policy <http://www.nature.com/info/privacy.html> | Update Profile <https://mts-cddis.nature.com>

Asunto: Re: CDDIS-21-4142RRR Initial Quality Check-URGENTE
De: CRISTINA NUNEZ DE ARENAS FLORES <cnunez@cbm.csic.es>
Fecha: 08/06/2022 16:12
Para: Laura Formentini <lformentini@cbm.csic.es>
CC: "JM. Cuezva" <jmcuezva@cbm.csic.es>, Laura Contreras Balsa <lcontreras@cbm.csic.es>, JUAN CRUZ HERRERO MARTIN <jherrero@cbm.csic.es>, Cristina Sánchez González <crissg31093@gmail.com>, Cristina Sánchez <cristina.sanchez@cbm.csic.es>

Yes, I agree with the final author list. Best regards, Cristina

Laura Formentini <lformentini@cbm.csic.es> escribió:

> As all of you already know, the work of Beñat Salegi Ansa, Brina Stancic and Marta P. Pereira was fundamental for answering Reviewer comments during revision.
> Cell Death and Disease request agreement from all authors for their addition into the final author list.
>
> Please, confirm that you agree to this final author list:
> "Sánchez-González, Cristina; Herrero Martín, Juan Cruz; Salegi Ansa, Beñat; Núñez de Arenas, Cristina; Stančič, Brina; Pereira P., Marta; Contreras, Laura; Cuezva, José M.; Formentini, Laura.^"
>
> Just replay to this email as your earliest convenience,
> Best regards,
> Laura
>
> -------- Mensaje reenviado --------
> Asunto: CDDIS-21-4142RRR Initial Quality Check
> Fecha: Wed, 8 Jun 2022 09:03:16 -0400
> De: cddisease@springernature.com
> Responder a: cddisease@springernature.com
> Para: lformentini@cbm.csic.es
>
> Dear Professor Formentini,
>
> In checking in your manuscript submitted to Cell Death & Disease it has come to our attention that the following must be addressed before we can proceed.
>
> 1. It has come to our attention that your most recent author list differs from the one in your original submission. We find that the following authors have been added since your initial submission: {Beñat Salegi Ansa; Brina Stančič; Marta P. Pereira}
>
> Please request agreement from all authors including additions and deletions, these can be collected in the following way:
>
> Email your co-authors with the change, and ask them to reply to your email confirming that they agree to these changes. Once you have collected these replies, please combine all of the co-authors' email responses in one document and upload this file to your submission.
>
> Your paper is now available for you to edit, you may access via the following link:
>
> https://mts-cddis.nature.com/cgi-bin/main.plex?el=A4CA2VyA1D5jSG5F3A9ftdsEGa8i7g6u5KZNVt5GaAZ
>
> (Press/Click on the above link to be automatically sent to the web page.)

Please make the correction(s) as specified above and resubmit your paper following the same steps as before.

If you have any questions please feel free to contact us.

Sincerely,

Editorial Office
Cell Death & Disease
cddisease@springernature.com

This email has been sent through the NPG Manuscript Tracking System NY-610A-NPG&MTS

/Confidentiality Statement:

This e-mail is confidential and subject to copyright. Any unauthorised use or disclosure of its contents is prohibited. If you have received this email in error please notify our Manuscript Tracking System Helpdesk team at http://platformsupport.nature.com . /

/Details of the confidentiality and pre-publicity policy may be found here http://www.nature.com/authors/policies/confidentiality.html /

Privacy Policy <http://www.nature.com/info/privacy.html> | Update Profile <https://mts-cddis.nature.com>

Asunto: Re: CDDIS-21-4142RRR Initial Quality Check-URGENTE
De: LAURA CONTRERAS BALSA <lcontreras@cbm.csic.es>
Fecha: 08/06/2022 16:01
Para: JUAN CRUZ HERRERO MARTIN <jherrero@cbm.csic.es>
CC: Laura Formentini <lformentini@cbm.csic.es>, Cristina Sánchez <cristina.sanchez@cbm.csic.es>, Cristina Sánchez González <crissg31093@gmail.com>, cnunez@cbm.csic.es, "JM. Cuezva" <jmcuezva@cbm.csic.es>

Yes, I agree with it.
Best regards,
L

JUAN CRUZ HERRERO MARTIN <jherrero@cbm.csic.es> escribió:

> I completely agree with the final author list.
>
> Best,
> Herrero Martín, Juan Cruz
>
> Laura Formentini <lformentini@cbm.csic.es> escribió:
>
>> As all of you already know, the work of Beñat Salegi Ansa, Brina Stancic and Marta P. Pereira was fundamental for answering Reviewer comments during revision.
>> Cell Death and Disease request agreement from all authors for their addition into the final author list.
>>
>> Please, confirm that you agree to this final author list:
>> "Sánchez-González, Cristina; Herrero Martín, Juan Cruz; Salegi Ansa, Beñat; Núñez de Arenas, Cristina; Stančič, Brina; Pereira P., Marta; Contreras, Laura; Cuezva, José M.; Formentini, Laura.^"
>>
>> Just replay to this email as your earliest convenience,
>> Best regards,
>> Laura
>>
>> -------- Mensaje reenviado --------
>> Asunto: CDDIS-21-4142RRR Initial Quality Check
>> Fecha: Wed, 8 Jun 2022 09:03:16 -0400
>> De: cddisease@springernature.com
>> Responder a: cddisease@springernature.com
>> Para: lformentini@cbm.csic.es
>>
>> Dear Professor Formentini,
>>
>> In checking in your manuscript submitted to Cell Death & Disease it has come to our attention that the following must be addressed before we can proceed.
>>
>> 1. It has come to our attention that your most recent author list differs from the one in your original submission. We find that the following authors have been added since your initial submission: {Beñat Salegi Ansa; Brina Stančič; Marta P. Pereira}
>>
>> Please request agreement from all authors including additions and deletions, these can be collected in the following way:
>>
>> Email your co-authors with the change, and ask them to reply to your email confirming that they agree to these changes. Once you have collected these replies, please combine all of the co-authors' email responses in one document and upload this file to your submission.

Your paper is now available for you to edit, you may access via the following link:

https://mts-cddis.nature.com/cgi-bin/main.plex?el=A4CA2VyA1D5jSG5F3A9ftdsEGa8i7g6u5KZNVt5GaAZ

(Press/Click on the above link to be automatically sent to the web page.)

Please make the correction(s) as specified above and resubmit your paper following the same steps as before.

If you have any questions please feel free to contact us.

Sincerely,

Editorial Office
Cell Death & Disease
cddisease@springernature.com

This email has been sent through the NPG Manuscript Tracking System NY-610A-NPG&MTS

/Confidentiality Statement:

This e-mail is confidential and subject to copyright. Any unauthorised use or disclosure of its contents is prohibited. If you have received this email in error please notify our Manuscript Tracking System Helpdesk team at http://platformsupport.nature.com . /

/Details of the confidentiality and pre-publicity policy may be found here http://www.nature.com/authors/policies/confidentiality.html /

Privacy Policy <http://www.nature.com/info/privacy.html> | Update Profile <https://mts-cddis.nature.com>

Asunto: Re: Fwd: CDDIS-21-4142RRR Initial Quality Check-URGENTE
De: JMCuezva <jmcuezva@cbm.csic.es>
Fecha: 08/06/2022 15:56
Para: Laura Formentini <lformentini@cbm.csic.es>, Cristina Sánchez <cristina.sanchez@cbm.csic.es>, Cristina Sánchez González <crissg31093@gmail.com>, JUAN CRUZ HERRERO MARTIN <jherrero@cbm.csic.es>, cnunez@cbm.csic.es, Laura Contreras Balsa <lcontreras@cbm.csic.es>

Yes, I agree with the final author list. Best, José

El 08/06/2022 a las 15:52, Laura Formentini escribió:

> As all of you already know, the work of Beñat Salegi Ansa, Brina Stancic and Marta P. Pereira was fundamental for answering Reviewer comments during revision.
> Cell Death and Disease request agreement from all authors for their addition into the final author list.
>
> Please, confirm that you agree to this final author list:
>
> "Sánchez-González, Cristina; Herrero Martín, Juan Cruz; Salegi Ansa, Beñat; Núñez de Arenas, Cristina; Stančič, Brina; Pereira P., Marta; Contreras, Laura; Cuezva, José M.; Formentini, Laura."
>
> Just replay to this email as your earliest convenience,
> Best regards,
> Laura
>
> -------- Mensaje reenviado --------
> **Asunto:** CDDIS-21-4142RRR Initial Quality Check
> **Fecha:** Wed, 8 Jun 2022 09:03:16 -0400
> **De:** cddisease@springernature.com
> **Responder a:** cddisease@springernature.com
> **Para:** lformentini@cbm.csic.es
>
> Dear Professor Formentini,
>
> In checking in your manuscript submitted to Cell Death & Disease it has come to our attention that the following must be addressed before we can proceed.
>
> 1. It has come to our attention that your most recent author list differs from the one in your original submission. We find that the following authors have been added since your initial submission: {Beñat Salegi Ansa; Brina Stančič; Marta P. Pereira}
>
> Please request agreement from all authors including additions and deletions, these can be collected in the following way:
>
> Email your co-authors with the change, and ask them to reply to your email confirming that they agree to these changes. Once you have collected these replies, please combine all of the

co-authors' email responses in one document and upload this file to your submission.

Your paper is now available for you to edit, you may access via the following link:

https://mts-cddis.nature.com/cgi-bin/main.plex?el=A4CA2VyA1D5jSG5F3A9ftdsEGa8i7g6u5KZNVt5GaAZ

(Press/Click on the above link to be automatically sent to the web page.)

Please make the correction(s) as specified above and resubmit your paper following the same steps as before.

If you have any questions please feel free to contact us.

Sincerely,

Editorial Office
Cell Death & Disease
cddisease@springernature.com

This email has been sent through the NPG Manuscript Tracking System NY-610A-NPG&MTS

Confidentiality Statement:

This e-mail is confidential and subject to copyright. Any unauthorised use or disclosure of its contents is prohibited. If you have received this email in error please notify our Manuscript Tracking System Helpdesk team at http://platformsupport.nature.com .

Details of the confidentiality and pre-publicity policy may be found here http://www.nature.com/authors/policies/confidentiality.html

Privacy Policy | Update Profile

--
Prof. José M. Cuezva
Centro de Biología Molecular "Severo Ochoa"
Universidad Autónoma de Madrid
28049 Madrid, SPAIN
Phone: 34.911.964.618
Fax: 34.911.964.420
Email: jmcuezva@cbm.csic.es or josem.cuezva@uam.es
http://www.cbmso.es/jmcuezva
